# Germline Genetic Variants of Viral Entry and Innate Immunity May Influence Susceptibility to SARS-CoV-2 Infection: Toward a Polygenic Risk Score for Risk Stratification

**DOI:** 10.3389/fimmu.2021.653489

**Published:** 2021-03-08

**Authors:** Vince Kornél Grolmusz, Anikó Bozsik, János Papp, Attila Patócs

**Affiliations:** ^1^Department of Molecular Genetics, National Institute of Oncology, Budapest, Hungary; ^2^Hereditary Tumors Research Group, Eötvös Loránd Research Network—Semmelweis University, Budapest, Hungary; ^3^Department of Laboratory Medicine, Semmelweis University, Budapest, Hungary

**Keywords:** SARS-CoV-2, COVID-19, genetic susceptibility, genotype-phenotype association studies, viral entry, innate immunity, polygenic risk score, risk stratification

## Abstract

The ongoing COVID-19 pandemic caused by the novel coronavirus, SARS-CoV-2 has affected all aspects of human society with a special focus on healthcare. Although older patients with preexisting chronic illnesses are more prone to develop severe complications, younger, healthy individuals might also exhibit serious manifestations. Previous studies directed to detect genetic susceptibility factors for earlier epidemics have provided evidence of certain protective variations. Following SARS-CoV-2 exposure, viral entry into cells followed by recognition and response by the innate immunity are key determinants of COVID-19 development. In the present review our aim was to conduct a thorough review of the literature on the role of single nucleotide polymorphisms (SNPs) as key agents affecting the viral entry of SARS-CoV-2 and innate immunity. Several SNPs within the scope of our approach were found to alter susceptibility to various bacterial and viral infections. Additionally, a multitude of studies confirmed genetic associations between the analyzed genes and autoimmune diseases, underlining the versatile immune consequences of these variants. Based on confirmed associations it is highly plausible that the SNPs affecting viral entry and innate immunity might confer altered susceptibility to SARS-CoV-2 infection and its complex clinical consequences. Anticipating several COVID-19 genomic susceptibility loci based on the ongoing genome wide association studies, our review also proposes that a well-established polygenic risk score would be able to clinically leverage the acquired knowledge.

## Introduction

Severe acute respiratory syndrome coronavirus-2 (SARS-CoV-2), the virus responsible for the ongoing pandemic COVID-19 has yet infected more than 108 million people worldwide with a reported mortality rate between 0.5 and 10% in different countries (1). SARS-CoV-2 is a novel coronavirus originally detected in China. The specific mechanism by which it infects humans and effects human health is not fully understood. The clinical characteristics of COVID-19 usually incorporates fever, fatigue, dry cough, and dyspnea, while severe infections may result in bilateral pneumonia, and life-threatening acute respiratory distress syndrome (ARDS). Although severe complications usually manifest in elder patients with concurrent chronic diseases (e.g., high blood pressure, diabetes) young, healthy individuals might also suffer from critical consequences of the disease, requiring intensive care. The wide range of disease susceptibility especially in younger patients suggests that difference in genetic background of individuals might contribute to these alterations. In fact, the analysis of previous, unrelated infectious diseases provides clear evidence that specific protective genetic variations are enriched in populations where certain infections are endemic. For instance, sickle cell trait and carrying specific HLA antigens in African populations confer diminished susceptibility against malaria infection (2, 3). Another example, Δ32, a 32-base pair deletion of the *CCR5* gene prevents cellular viral entry of human immunodeficiency virus (HIV) resulting in effective resistance against HIV infection in individuals homozygous regarding this variation (4).

In the present review we aim to summarize previously published genotype-phenotype studies of genes which might play a role in the susceptibility to COVID-19. The associations between various single nucleotide polymorphisms (SNPs) and certain traits were studied using targeted and genome-wide approaches. In the case of targeted approach, hypothesis-driven selection of specific genes/SNPs were analyzed in cases and controls while during genome-wide association studies (GWASs) detection of novel genomic loci with susceptibility to various traits/diseases are possible. Our examination focuses on genetic variants of 2 key processes in the initiation of the disease: viral entry and recognition and response by the innate immune system. Also, as several international collaborations are ongoing to provide large-scale genomic susceptibility data, we propose that a well-established polygenic risk score would be able to optimally leverage the acquired knowledge.

## Viral Entry

Large emphasis has been directed to decipher how SARS-CoV-2 is incorporated in human cells. Key data in this regard originate from studies focusing on SARS-CoV, responsible for the SARS epidemic of 2002–2003, which shares 79.6% sequence identity with SARS-CoV-2 (5). In fact, the spike protein of SARS-CoV binds to angiotensin-converting enzyme 2 (ACE2) that serves as a receptor for the virus (6), and recent data confirmed that SARS-CoV-2 also binds ACE2 *in vitro* (7–9). Further analyses revealed that the spike protein of SARS-CoV-2 is cleaved by transmembrane protease serine 2 (TMPRSS2) (7), facilitating viral entry. Also of note, both ACE2 and TMPRSS2 are primarily expressed in bronchial transient secretory cells (10), elucidating the predilection of the lower airways. Additionally, proprotein convertase FURIN was shown to pre-activate the viral entry of SARS-CoV-2 (11), while additional factors as PIKfyve, *TPCN2* and cathepsin L (*CTSL*) are also critical in this process (12).

Table 1 summarizes genetic variants of the aforementioned genes with suggested genotype-phenotype findings. The main physiological function of ACE2 is catalyzing the hydrolysis of angiotensin I and angiotensin II into angiotensin (1–9) and angiotensin (1–7), respectively, contributing to blood pressure regulation (34). Therefore, numerous SNP association studies were directed to ascertain the role of *ACE2* genetic variants on certain cardiovascular and metabolic traits. Throughout several populations, *ACE2* polymorphisms have been associated with susceptibility to cardiovascular and metabolic diseases including hypertension and type 2 diabetes mellitus underlining the potential functional impact of these SNPs on *ACE2* expression and/or function (13–21). A *TMPRSS2* SNP has been linked to *TMPRSS2-ERG* genetic fusion which is a frequent molecular event in prostate cancer (22, 23). More importantly, a study examining patients of the 2009 swine flu pandemic caused by the H1N1 influenza virus found that *TMPRSS2* SNP rs2070788 is associated with severity of the disease (24). Additionally, genotype-specific *TMPRSS2* expression was confirmed in human lung tissues regarding rs2070788 and rs383510, the latter being tagged to the former polymorphism. Mechanistically, rs383510 was found to enhance the transcription of TMPRSS2 mRNA, and these 2 SNPs were also found to associate with susceptibility to the H7N9 influenza virus (24).

**Table 1:** Genotype-phenotype associations of genes involved in viral entry of SARS-CoV-2.

**Chromosome**	**Gene ID**	**Transcript ID**	**Gene**	**SNP**	**MAF**	**Position**	**Exon(E)/intron(I)**	**Observed association**			
								**Trait type**	**Trait**	**Population**	**References**
X	ENSG00000130234	ENST00000427411.1	*ACE2*	rs2074192	0.36	15564667	I17–18	O	Left ventricular hypertrophy (LVH) in females	Chinese Han	(13)
								O	T2DM	Uygur	(14)
								O	Diabetic retinopathy within female T2DM patients	Chinese	(15)
				rs4646176	0.07	15569381	I15–16	O	Essential hypertension (EH) in females	Northeastern Chinese Han	(16)
				rs4646155	0.06	15579386	I9–10	O	Essential hypertension (EH) in females	Northeastern Chinese Han	(16)
				rs2106809	0.32	15599938	I2–3	O	Left ventricular hypertrophy (LVH) in females	Chinese Han	(13)
								O	Lone atrial fibrillation	Chinese	(17)
				rs1514283	0.11	15564624	I17–18	O	Essential hypertension (EH) in females	Northeastern Chinese Han	(16)
				rs2285666	0.35	15592225	I4–5	O	Essential hypertension (EH) in females	Northeastern Chinese Han	(16)
								O	Cardiovascular death in females	European	(18)
				rs879922	0.32	15572684	I12–13	O	Essential hypertension (EH) in females	Northeastern Chinese Han	(16)
								O	T2DM	Uygur	(14)
				rs1978124	0.21	15599940	I2–3	O	T2DM	Uygur	(14)
				rs2048683	0.20	15590376	I5–6	O	T2DM	Uygur	(14)
				rs233575	0.14	15564843	I17–18	O	T2DM	Uygur	(14)
				rs4240157	0.32	15568841	I15–16	O	T2DM	Uygur	(14)
				rs4646156	0.20	15578920	I9–10	O	T2DM	Uygur	(14)
				rs4646188	0.04	15583220	I8–9	O	T2DM	Uygur	(14)
				rs6632677	0.02	15596749	I2–3	O	Structural atrial fibrillation in males	Chinese Han	(19)
								I/A	Dilated cardiomyopathy (DCM)	North Indian	(20)
				rs714205	0.31	15565781	I17–18	O	Diabetic retinopathy within female T2DM patients	Chinese	(15)
				rs4646174	0.32	15570148	I15–16	O	Blood pressure responses after potassium supplementation in males	Chinese Han	(21)
21	ENSG00000184012	ENST00000332149.10	*TMPRSS2*	rs12329760	0.26	41480570	E6	N	TMPRSS2-ERG fusion in patients with prostate cancer	Indian	(22)
								N	TMPRSS2-ERG fusion by translocation, multiple copies of the gene fusion	American	(23)
				rs2070788	0.40	41470061	I11–12	I	Severe H1N1 infection, H7N9 infection	Chinese	(24)
				rs383510	0.40	41486440	I5–6	I	Severe H1N1 infection, H7N9 infection	Chinese	(24)
15	ENSG00000140564	ENST00000268171.8	*FURIN*	rs4702	0.35	90883330	E16	O	Systolic and diastolic blood pressure	Finnish	(25)
								O	Schizophrenia	American	(26)
				rs17514846	0.47	90873320	I1–2	O	Coronary artery disease	N.A. (metaanalyis)	(27)
								O	Metabolic syndrome	Japanese	(28)
								O	Longevity, parents' attained age	European	(29)
11	ENSG00000162341	ENST00000294309.8	*TPCN2*	rs1551305	0.34	69087765	I24–25	O	T2DM	Chinese	(30)
				rs35264875	0.10	69078931	E16	O	Hair color (blond vs. brown)	European	(31)
				rs3829241	0.18	69087895	E25	O	Hair color (blond vs. brown)	European	(31)
9	ENSG00000135047	ENST00000343150.10	*CTSL*	rs3118869	0.43	87725948	5' upstream	O	Essential hypertension (EH)	Uygur, Kazak and Han Chinese	(32)
								O	Hypertension, systolic blood pressure, diastolic blood pressure	American	(33)

*Regarding locus specifications genome build GRCh38.p13 was used and for minor allele frequency (MAF) of the second most frequent allele in 1,000 Genomes Phase three combined population is demonstrated, where available. SNP, single nucleotide polymorphism; MAF, minor allele frequency; T2DM, type 2 diabetes mellitus. A, autoimmune; I, infectious; N, neoplastic; O, other*.

Certain high throughput screening studies identified rs4702, a common genetic variant of proprotein convertase *FURIN* as susceptibility factor for schizophrenia and hypertension (25, 26), while other studies correlated another SNP rs17514846 with other various traits including coronary artery disease, metabolic syndrome and longevity (27–29).

While we found no SNP association studies for PIKfyve, certain variants of the *TPCN2* gene coding for cation-selective ion channel were found to be associated with type 2 diabetes mellitus (T2DM) and hair color (30, 31). In the case of *CTSL*, two studies performed on different populations confirmed that a promoter polymorphism correlates with hypertension in Asian and American populations (32, 33).

## Innate Immunity

After SARS-CoV-2 successfully infected cells, a complex immune response initiates, in which the rapid and coordinated response of the innate immunity is pre-requisite (35). Following infection, the innate immune system recognizes viral antigens mainly by RIG-I-Like Receptors (RLRs) and Toll-Like Receptors (TLRs) (35). In the first step in RLR-dependent immune response, cytoplasmic RNA sensors RIG-I and MDA5 recognize viral RNA, after which interaction with mitochondrial antiviral signaling protein (MAVS) initiate signaling changes activating interferon regulatory factor *IRF3* and *IRF7*, resulting in type I IFN (IFN-α and IFN-β) production and antiviral response (35–38).

Supplementary Table 1 summarizes the SNP association studies concerning the agents implicated in viral recognition and response by the innate immune system. Several RIG-I SNPs were found to be associated with neutralizing antibody levels after measles and rubella vaccinations while other studies found RIG-I SNPs to be associated with nasopharyngeal carcinoma and EV71-induced hand, foot, and mouth disease (39–43). *MDA5* genetic variants were thoroughly investigated in relation to autoimmunity with several associations being found with psoriasis, systemic lupus erythematosus (SLE), type 1 diabetes mellitus (T1DM), hypothyroidism and multiple sclerosis (MS) (44–53). Polymorphisms in *MAVS* were analyzed regarding inflammatory response finding that rs7269320 associated with osteoarthritis (54). Moreover, in an African American cohort, where rs11905552 of the *MAVS* gene was much more frequent compared to European Americans, this SNP associated with low type I IFN production in patients with SLE (55). Studies focusing on genetic variants of *IRF3* and *IRF7* found associations with SLE and systemic sclerosis (56–59), while IFN-α genetic variants were found to be associated with mixed connective tissue disease and prognosis in glioma patients (60, 61).

During TLR-mediated immune response, TLR3, TLR7, TLR8, and TLR9 sense intracellular, while TLR2 and TLR4 detect extracellular, cell surface-associated viral antigens (35, 62). TLRs transduce the signal by binding MyD88 and TRIF, which in turn stimulate IRF3, IRF7, and NF-κB enhancing type I IFN response (35, 63–65).

A multitude of case-control studies analyzed the role of TLR-associated SNPs in health and disease. *TLR3* SNPs were associated with infectious, autoimmune, and neoplastic diseases. Certain studies demonstrated association of *TLR3* polymorphisms with hepatitis B and C virus (HBV and HCV), herpes simplex virus (HSV), HIV infections (66–72), while SNPs rs3775291, rs3775292, rs5743312, and rs7657186 were associated with vaccine-induced immunity to serogroup C meningococcal vaccine as defined by virus-specific IgG persistence (73). rs3775291 was also associated with various autoimmune disorders including SLE, rheumatoid arthritis (RA), and sarcoidosis (74–76). Regarding neoplastic diseases, *TLR3* genetic variants were linked to breast, colorectal and nasopharyngeal cancers, while also serving as prognostic factors in colorectal cancer (CRC) and melanoma malignum (MM) (42, 77–81). Several lines of evidence supported the association between SLE and *TLR7* SNPs in various populations (82–85), while additional studies found correlation between *TLR7* polymorphisms and susceptibility to HCV and chikungunya virus infection, asthma, and age-related macular degeneration (86–89). The association between rs3764880 of *TLR8* and tuberculosis susceptibility in males has been confirmed in European, Russian, and Chinese populations (90–92) and other SNPs of *TLR8* were associated with asthma, SLE, and chikungunya virus infection (84, 87, 88). With regard to the fourth TLR sensing intracellular viral antigens, *TLR9* has 4 SNPs which were found to be associated with several infectious, autoimmune, and neoplastic diseases. Confirmed associations with infectious diseases include malaria, cytomegalovirus (CMV), and tuberculosis (90, 93–97), while individuals with certain *TLR9* polymorphisms are more susceptible to post-infectious irritable bowel syndrome, SLE and lupus nephritis, Graves' disease-related ophthalmopathy, and RA (98–104). With respect to neoplastic diseases, acute myeloid leukemia (AML), and cervical cancer were also associated with *TLR9* genetic variants (105–107), while rs187084 is proposed to be a prognostic factor in patients with prostate cancer (108).

*TLR2* and *TLR4* SNPs are probably the most widely investigated genetic variants in the scope of our review. Similarly to studies conducted in other TLR genes, *TLR2* polymorphisms were also found to be associated with tuberculosis and CMV infection (90, 109–111), and additional pathogenic role concerning bacterial vaginosis in HIV-infected patients, recurrent vulvovaginal candidiasis, aggressive periodontitis, neonatal sepsis, Lyme disease, pneumonia, and reactive arthritis were also proposed (112–119). SNP rs3804100 has been linked to measles-specific antibody levels following immunization, while rs5743708 associated with nasal Staphylococcus aureus carriage (120, 121). Autoimmune disorders linked to *TLR2* SNPs incorporate psoriasis and T1DM (122, 123), while hepatocellular carcinoma (HCC), marginal zone lymphoma, oral, and laryngeal squamous cell carcinoma and prognosis of women with breast cancer have also been linked to certain genetic variants of *TLR2* (124–127).

*TLR4* polymorphisms have been associated with various infectious diseases. Manifest tuberculosis is associated with rs11536889, rs12377632, rs1927911, and rs7873784 (109, 110, 128), while additional associated infection-related diseases include sepsis and sepsis-related organ failure for rs11536889 and Chlamydia trachomatis infection in women with pelvic inflammatory disease for rs1927911 (129–131). The most intensively investigated *TLR4* SNP, rs4986790 is associated with a wide range of infections including Gram-negative and Mycobacterium bacteria in high-risk populations, severe respiratory syncytial virus disease, clinical malaria, recurrent cystitis, chronic cavitary pulmonary aspergillosis, HCV infection, and prognosis of HBV-infected individuals (132–141). It also has a probable effect on IL-4 secretion after measles vaccination (142). Another SNP of *TLR4*, rs5030717 is associated with childhood otitis media (143).

With respect to autoimmune-related diseases, *TLR4* SNPs associate with ankylosing spondylitis, RA, giant cell arteritis, and preeclampsia (144–147). In addition, rs10759932 and rs4986790 are linked to acute rejection following kidney and lung transplantation, respectively (148, 149). Several *TLR4* polymorphisms (rs10759932, rs10983755, rs11536889, rs1927911, rs2149356, and rs4986790) are associated with gastric cancer susceptibility, where the risk-elevating Helicobacter pylori infection might have an important role (150–154). Other tumors linked to *TLR4* genetic variants include HCC, prostate cancer, CRC, and non-Hodgkin lymphoma (NHL) (79, 155–160).

Genetic variants of adapter molecule MYD88 are associated with tuberculosis susceptibility, Buerger disease and treatment response in patients with RA (90, 161–163). SNPs of the other key adapter agent, TRIF are associated with pneumonia susceptibility and thyroid cancer (164, 165).

In addition to type I IFN response viral recognition in the innate immune system leads to NF-kB activation. NF-kB is a multiprotein complex consisting of NFKB1, NFKB2, RELA, RELB, and REL (166). Type I IFN response and NF-kB activation result in IL-6 and IL-8 production (35). The activation of these mediators contributes to inflammation and complex antiviral immune response (35).

As a key player in inflammatory response, *NFKB1* genetic variants has also been associated with atherosclerotic manifestations including coronary artery disease, acute coronary syndrome, dilated cardiomyopathy, and ischaemic stroke (167–173). Promoter polymorphism rs28362491 is linked to HCV infection and autoimmune diseases including Behcet's disease and SLE (174–176), while rs3774937 is associated with acute rejection after renal transplantation (177). Neoplastic diseases associated with *NFKB1* SNPs include CRC, Hodgkin lymphoma, NHL, cervical squamous cell carcinoma, liver, thyroid, breast, and lung cancer (178–186). rs11574851 of *NFKB2* was found to be linked to RA susceptibility among anti-citrullinated protein antibodies-positive patients (187), while in healthy women rs1049728 of *RELA* associated with the concentration of soluble ICAM-1, which is an endothelium-derived inflammatory marker (188). Genetic variants of *REL* have been shown to be linked to various autoimmune diseases including RA, psoriasis, and celiac disease (189–193).

Polymorphisms of *IL6* have been shown to pre-dispose to pulmonary tuberculosis, acute lung injury in patients with systemic inflammatory response syndrome and post-infectious irritable bowel syndrome (98, 194, 195). An association with RA has also been proposed (196). rs1800795 has been shown to have a role in the prognosis of patients following renal and lung transplantation (197–199). *IL6* SNPs were also confirmed to have a role in the susceptibility of various cardiovascular disorders including hypertension and stroke (200–202).

IL-8 is coded by *CXCL8* gene, SNPs of which have been shown to be linked to infectious, autoimmune, and neoplastic diseases. Acne vulgaris, chronic periodontitis, and invasive aspergillosis among immunocompromised patients have been shown to be associated with various variants (203–205). Autoimmune diseases including idiopathic pulmonary fibrosis, childhood IgA nephropathy, erosive oral lichen planus, childhood asthma, and Graves' disease have also been linked to genetic variants of *CXCL8* (206–210). Concerning neoplastic diseases, non-small cell lung cancer, and gastric cancer have been proposed to be associated with *CXCL8* SNPs (154, 211, 212).

In conclusion, large majority of the discussed SNPs present pleiotropic effects, among which the frequent presence of various autoimmune and infection-related traits highlights their putative involvement in the susceptibility and severity of COVID-19.

## Toward Precision Risk Assessment: Predicting COVID-19 Susceptibility and Severity Based on a Polygenic Risk Score

As genetic susceptibility regarding COVID-19 is an ongoing topic of several large international collaborations we anticipate to acquire a large amount of evidence regarding susceptibility loci in the near future. Indeed, recent studies identified germline variants of TLR3- and IRF7-dependent type I IFN immunity to associate with more severe COVID-19 infection (213). In particular, disease-causing germline variants have been detected in *TLR3, UNC93B1, TICAM1, TBK1, IRF3, IRF7, IFNAR1*, and *IFNAR2* in patients with life-threatening COVID-19 (213). Another recent study analyzing 1,610 COVID-19 patients and 2,205 control subjects from the first wave in heavily affected Italy and Spain found 2 chromosomal loci on chromosome three and nine with significant association with COVID-19 patients (214). On chromosome three the affected area includes several actors which might alter COVID-19 susceptibility including chemokine receptors, while on chromosome nine the association signal coincided with the AB0 blood group locus (214). AB0 blood group has independently been linked to COVID-19 susceptibility (214–216). Further studies are needed to confirm these associations in independent populations.

**Figure 1:**
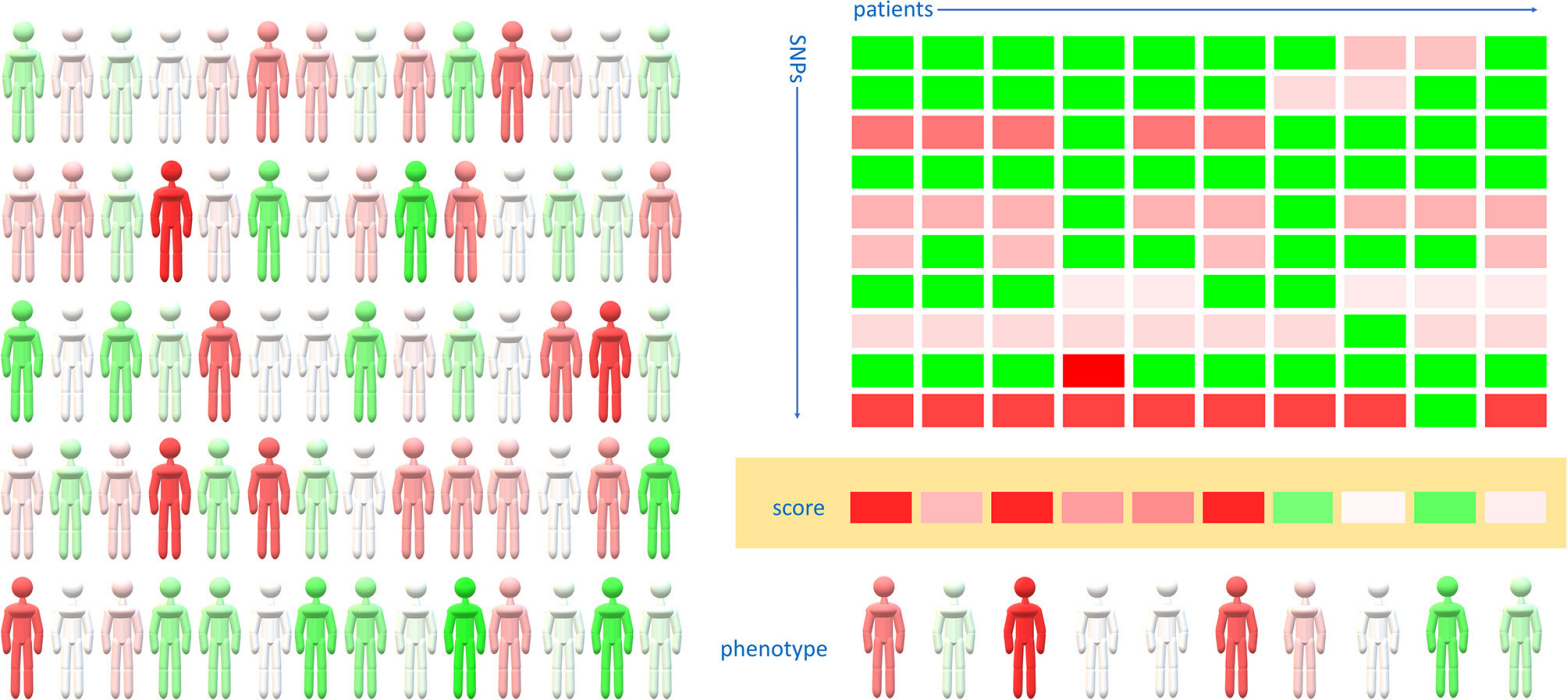
Polygenic risk scores might detect high-risk individuals regarding COVID-19 susceptibility and severity. The actual susceptibility and severity of COVID-19 varies widely within the population (left panel, redder individuals are more, greener individuals are less prone for severe COVID-19 disease). Genome-wide association studies might distinguish a group of SNPs from which a clinically relevant polygenic risk score can be built (right panel). Color-coded squares represent the presence of the risk allele (red) or the alternative allele (green) in each individual. The intensity of red corresponds to the odds ratio of the risk allele compared to the alternative allele. Resultant values of the odds ratios of each SNPs are color-coded as the polygenic risk score (orange background). Personalized risk scores correlate well with actual COVID-19 risk, however additional environmental, anthropometric factors and comorbidities also modify the phenotype.

Applying this knowledge to detect individuals with elevated risk for severe disease might help to prioritize them for vaccination and stricter protection measures. As COVID-19 susceptibility and severity seem to have a polygenic background, we propose that a curated polygenic risk score (PGRS) might facilitate the detection of individuals with high risk for infection (Figure 1). Based on genome-wide analyses, polygenic risk scores are able to detect high-risk individuals in various diseases, fine-tuning the more widely used risk stratification dependent on baseline anthropometric and physiological characteristics (217, 218). A most recent GWAS on a cohort of COVID-19 patients from the U.K. found eight lead variants from independent genome-wide significant regions including rs2236757 in *IFNAR2* coding for interferon α and β receptor subunit 2 (219). Though the individual odds ratio for each of the relatively frequent variants varies between 1.3 and 2.1, the combined odds ratio in the case of harboring all these susceptibility variants rises to 29.5, underlining the applicability of a polygenic risk score (219).

In addition to COVID-19 susceptibility, inclusion of genetic predictors of disease severity and treatment response might also be included. In particular, based on the effectiveness of glucocorticoid administration confirmed by the randomized, controlled RECOVERY clinical trial (220), it would be interesting to see if pharmacogenetic modifiers of glucocorticoid action, sensitivity and metabolism contribute to the severity of COVID-19 infection and treatment response (221).

It is important to note that the majority of the observed associations in Table 1 and Supplementary Table 1 were only validated in specific populations. By analyzing the population-specific allelic frequencies of the reviewed viral entry and innate immunity-related SNPs reviewed (Supplementary Table 2) we can conclude that the large variations in SNP frequencies might heavily influence their association with various traits in select populations. Additionally, pronounced differences in risk allele frequencies of the 8 proposed lead COVID-19-related SNPs (219) are present in different populations (Supplementary Table 3). Moreover, these differences most probably alter epistatic interactions between genes, adding an additional layer of complexity (222).

Therefore, the observed population dependency of genotype-phenotype associations would probably result in population-specific PGRSs rather than a universal PGRS optimal for all populations. Dedicated efforts to perform population-specific GWASs regarding COVID-19 susceptibility and severity to build population-specific PGRSs are needed to address these differences.

## Discussion

The disruption caused by the COVID-19 pandemic has yet unknown consequences on the whole human society and on each affected patient's health as well. Understanding the susceptibility toward this disease is important to detect high-risk individuals and also to decipher molecular mechanisms needed for the development of the clinical phenotype. Viral entry and innate immunity are key mechanisms in the initiation of SARS-CoV-2 infection. We performed a thorough literature review concerning genotype-phenotype association studies regarding agents of these mechanisms. Our results indicated that SNPs in the genes of these processes are frequently associated with susceptibility to various bacterial and viral infections. Additionally, several autoimmune diseases are also linked to these genes, underlining the versatile immune consequences of these genetic variants. Based on the confirmed associations it is highly plausible that the abovementioned SNPs might confer altered susceptibility to SARS-CoV-2 infection and its complex clinical consequences.

In addition to viral entry and innate immunity, other mechanisms including adaptive immunity are also of paramount importance regarding the susceptibility to COVID-19 (35). To better characterize putative genomic susceptibility loci, well-designed, international genome-wide association studies (GWAS) are needed.

As multiple GWASs on host genetic susceptibility are ongoing, several genomic susceptibility loci are proposed to be detected. Translating these individual susceptibility variants into clinically relevant polygenic risk scores would fully leverage this acquired knowledge to easily detect high-risk individuals prioritized for vaccination and stricter protective measures.

All things considered, genetic variants of genes of viral entry and innate immunity might alter susceptibility, and prognosis of COVID-19. Further GWASs are needed to better characterize susceptibility loci and to develop clinically relevant risk stratification strategies.

## Author Contributions

VG contributed to the design, performed literature search, and drafted the manuscript. AB contributed to the literature search. JP contributed to the design and the literature search. AP conceived the review, contributed to the design, and literature search. All authors read and have agreed to the final manuscript.

## Conflict of Interest

The authors declare that the research was conducted in the absence of any commercial or financial relationships that could be construed as a potential conflict of interest.
