# Supplementary material for: Germline Genetic Variants of Viral Entry and Innate Immunity May Influence Susceptibility to SARS-CoV-2 Infection: Toward a Polygenic Risk Score for Risk Stratification
Source: Front Immunol. 2021 Mar 8;12:653489. doi: 10.3389/fimmu.2021.653489 (PMC7982482; doi:10.3389/fimmu.2021.653489)
Supplement: Supplementary file 1 [file Table_1.docx]

Supplementary Material

# Supplementary Table 1

**Genotype-phenotype associations of genes involved in the innate immunity** Note: Regarding locus specifications genome build GRCh38.p13 was used and for minor allele frequency (MAF) of the second most frequent allele in 1000 Genomes Phase 3 combined population is demonstrated, where available. Gene names in bold correspond to confirmed association with COVID-19 susceptibility. Abbreviations: SNP: single nucleotide polymorphism, MAF: minor allele frequency, T2DM: type 2 diabetes mellitus, T1DM: type 1 diabetes mellitus, SLE: systemic lupus erythematosus, IFN: interferon, HBV: hepatitis B virus, HC: hepatitis C virus, HIV: human immunodeficiency virus, CMV: cytomegalovirus, HELLP: Hemolysis, elevated liver enzymes, low platelet count. Abbreviations for trait type column: A: autoimmune, I: infectious, N: neoplastic, O: other.

| chromosome | gene ID | transcript ID | gene | SNP | MAF | position | exon(E)/intron(I) | observed association | | | |
| --- | --- | --- | --- | --- | --- | --- | --- | --- | --- | --- | --- |
|  |  |  |  |  |  |  |  | trait type | trait | population | reference |
| 9 | ENSG00000107201 | ENST00000379883.3 | *RIGI/DDX58* | rs10813831 | 0.18 | 32526148 | E1 | I | decreased rubella antibody level following rubella vaccination | American | ([1](#_ENREF_1)) |
|  |  |  |  |  |  |  |  | I | modulates HCV infection severity | American | ([2](#_ENREF_2)) |
|  |  |  |  | rs669260 | 0.10 | 32503442 | I1-2 | I | increased neutralizing antibody titers for both measles and rubella viral antigens post-MMR vaccination | American | ([3](#_ENREF_3)) |
|  |  |  |  | rs3739674 | 0.46 | 32526235 | 5' upstream | I | increased risk of EV71-induced hand, foot, and mouth disease | Han Chinese | ([4](#_ENREF_4)) |
|  |  |  |  | rs3205166 | 0.31 | 32459452 | E17 | I | measles virus-specific neutralizing antibody level | Caucasian American | ([5](#_ENREF_5)) |
|  |  |  |  | rs11795343 | 0.35 | 32523739 | I1-2 | I | measles virus-specific neutralizing antibody level | Caucasian American | ([5](#_ENREF_5)) |
|  |  |  |  | rs56309110 | 0.18 | 32526756 | 5' upstream | N | nasopharyngeal carcinoma | North African | ([6](#_ENREF_6)) |
| 2 | ENSG00000115267 | ENST00000649979.2 | *MDA5/IFIH1* | rs10930046 | 0.19 | 162281473 | E7 | A | decreased risk for psoriasis | Caucasian American | ([7](#_ENREF_7)) |
|  |  |  |  |  |  |  |  | A | decreased risk for SLE | African American | ([8](#_ENREF_8)) |
|  |  |  |  | rs13023380 | 0.22 | 162297853 | I3-4 | A | decreased risk for SLE | African American and European American | ([8](#_ENREF_8)) |
|  |  |  |  | rs1990760 | 0.36 | 162267541 | E15 | A | decreased risk for SLE | African American and European American | ([8](#_ENREF_8)) |
|  |  |  |  |  |  |  |  | A | T1DM | Hungarian | ([9](#_ENREF_9)) |
|  |  |  |  |  |  |  |  | A | T1DM | British | ([10](#_ENREF_10)) |
|  |  |  |  | rs35337543 | 0.01 | 162279995 | I8-9 | O | hypothyroidism | UK population | ([11](#_ENREF_11)) |
|  |  |  |  |  |  |  |  | A | decreased risk for T1DM | British | ([12](#_ENREF_12)) |
|  |  |  |  | rs35667974 | <0.01 | 162268127 | E14 | A | decreased risk for psoriasis | Caucasian American | ([7](#_ENREF_7)) |
|  |  |  |  |  |  |  |  | A | decreased risk for T1DM | Russian | ([13](#_ENREF_13)) |
|  |  |  |  |  |  |  |  | A | decreased risk for T1DM | British | ([12](#_ENREF_12)) |
|  |  |  |  | rs35744605 | <0.01 | 162277580 | E10 | A | decreased risk for T1DM | Russian | ([13](#_ENREF_13)) |
|  |  |  |  | rs3747517 | 0.41 | 162272314 | E13 | A | multiple sclerosis | Danish | ([14](#_ENREF_14)) |
|  |  |  |  |  |  |  |  | A | T1DM | Han Chinese | ([15](#_ENREF_15)) |
|  |  |  |  |  |  |  |  | A | SLE | Swedish | ([16](#_ENREF_16)) |
|  |  |  |  | rs78456138 | <0.01 | 162275836 | I11-12 | A | SLE | Swedish | ([16](#_ENREF_16)) |
| 20 | ENSG00000088888 | ENST00000428216.4 | *MAVS* | rs7269320 | 0.21 | 3865750 | E7 | O | osteoarthritis | Han Chinese | ([17](#_ENREF_17)) |
|  |  |  |  | rs11905552 | 0.04 | 3857753 | E3 | A | low type I IFN production in SLE patients | African American | ([18](#_ENREF_18)) |
| 19 | ENSG00000126456 | ENST00000377139.8 | ***IRF3*** | rs2304204 | 0.40 | 49665763 | E1 | N | colon cancer | American | ([19](#_ENREF_19)) |
|  |  |  |  |  |  |  |  | A | decreased risk for SLE | Mexican Mestizo | ([20](#_ENREF_20)) |
|  |  |  |  | rs2304206 | 0.37 | 49665614 | I1-2 | A | increased risk for SLE | Mexican Mestizo | ([20](#_ENREF_20)) |
| 11 | ENSG00000185507 | ENST00000525445.6 | ***IRF7*** | rs1061501 | 0.18 | 614864 | E4 | A | decreased risk for SLE | Taiwanese | ([21](#_ENREF_21)) |
|  |  |  |  | rs1131665 | 0.28 | 613208 | E9 | A | decreased risk for SLE | Asian, Chinese, European American | ([22](#_ENREF_22)) |
|  |  |  |  |  |  |  |  | A | decreased risk for systemic sclerosis (SS) | Caucasian | ([23](#_ENREF_23)) |
|  |  |  |  | rs3758650 | 0.06 | 616865 | 5' upstream | O | gallstone disease | Taiwanese | ([24](#_ENREF_24)) |
| 9 | ENSG00000147873 | ENST00000610521.1 | *IFNA5* | rs3758236 | 0.25 | 21307785 | 5' upstream | A | mixed connective tissue disease | Polish | ([25](#_ENREF_25)) |
| 9 | ENSG00000120242 | ENST00000380205.2 | *IFNA8* | rs12553612 | 0.06 | 21408858 | 5' upstream | N | survival of glioma patients | Caucasian American | ([26](#_ENREF_26)) |
| 4 | ENSG00000164342 | ENST00000296795.8 | ***TLR3*** | rs10025405 | 0.40 | 186085652 | E5 | N | breast cancer in females | African American | ([27](#_ENREF_27)) |
|  |  |  |  | rs11721827 | 0.11 | 186069983 | I1-2 | N | rectal cancer | American | ([28](#_ENREF_28)) |
|  |  |  |  | rs13126816 | 0.21 | 186073024 | I1-2 | I | herpes simplex virus type 2 infection | Swedish | ([29](#_ENREF_29)) |
|  |  |  |  |  |  |  |  | I | chronic HCV infection | American | ([30](#_ENREF_30)) |
|  |  |  |  | rs1879026 | 0.16 | 186079167 | I3-4 | I | hepatitis B virus infection | Saudi Arabian | ([31](#_ENREF_31)) |
|  |  |  |  |  |  |  |  | O | severe hepatitis in newborns | Chinese | ([32](#_ENREF_32)) |
|  |  |  |  | rs3775290 | 0.27 | 186083063 | E4 | I | chronic hepatitis B infection | Chinese | ([33](#_ENREF_33)) |
|  |  |  |  |  |  |  |  | O | early onset Parkinson disease | Han Chinese | ([34](#_ENREF_34)) |
|  |  |  |  | rs3775291 | 0.23 | 186082920 | E4 | O | geographic atrophy subtype of age-related macular degeneration | European Americans | ([35](#_ENREF_35)) |
|  |  |  |  |  |  |  |  | N | colorectal cancer-specific survival | German | ([36](#_ENREF_36)) |
|  |  |  |  |  |  |  |  | I | HIV infection | Spanish and Italian | ([37](#_ENREF_37)) |
|  |  |  |  |  |  |  |  | I | vaccine-induced immunity to serogroup C meningococcal vaccine | British | ([38](#_ENREF_38)) |
|  |  |  |  |  |  |  |  | I | subacute sclerosing panencephalitis | Japanese | ([39](#_ENREF_39)) |
|  |  |  |  |  |  |  |  | I | herpes simplex virus type 2 infection | Swedish | ([29](#_ENREF_29)) |
|  |  |  |  |  |  |  |  | N | nasopharyngeal carcinoma | North African | ([6](#_ENREF_6)) |
|  |  |  |  |  |  |  |  | I | HIV infection in highly exposed intravenous drug users | Caucasian Estonian | ([40](#_ENREF_40)) |
|  |  |  |  |  |  |  |  | A | SLE in females | Danish | ([41](#_ENREF_41)) |
|  |  |  |  |  |  |  |  | A | rheumatoid arthritis | Danish | ([42](#_ENREF_42)) |
|  |  |  |  |  |  |  |  | I/A | sarcoidosis | Japanese | ([43](#_ENREF_43)) |
|  |  |  |  | rs3775292 | 0.17 | 186081871 | I3-4 | N | colon cancer | American | ([28](#_ENREF_28)) |
|  |  |  |  |  |  |  |  | I | vaccine-induced immunity to serogroup C meningococcal vaccine | British | ([38](#_ENREF_38)) |
|  |  |  |  | rs5743305 | 0.32 | 186068179 | 5' upstream | N | breast cancer in females | Chinese | ([44](#_ENREF_44)) |
|  |  |  |  | rs5743312 | 0.15 | 186079102 | I3-4 | I | vaccine-induced immunity to serogroup C meningococcal vaccine | British | ([38](#_ENREF_38)) |
|  |  |  |  | rs7657186 | 0.21 | 186072885 | I1-2 | I | vaccine-induced immunity to serogroup C meningococcal vaccine | British | ([38](#_ENREF_38)) |
|  |  |  |  | rs7668666 | 0.23 | 186080138 | I3-4 | N | prognosis in melanoma patients | Caucasian American | ([45](#_ENREF_45)) |
|  |  |  |  | rs78726532 | 0.04 | 186079310 | I3-4 | I | HCV infection | Saudi Arabian | ([46](#_ENREF_46)) |
| X | ENSG00000196664 | ENST00000380659.4 | *TLR7* | rs179008 | 0.12 | 12885540 | E3 | O | age-related macular degeneration in females | American | ([47](#_ENREF_47)) |
|  |  |  |  |  |  |  |  | A | athma in boys | Danish | ([48](#_ENREF_48)) |
|  |  |  |  | rs3853839 | 0.40 | 12889539 | E3 | A | SLE in females | Japanese | ([49](#_ENREF_49)) |
|  |  |  |  |  |  |  |  | A | SLE | Eastern Asian | ([50](#_ENREF_50)) |
|  |  |  |  |  |  |  |  | A | SLE | Danish | ([51](#_ENREF_51)) |
|  |  |  |  |  |  |  |  | A | SLE in females | Egyptian | ([52](#_ENREF_52)) |
|  |  |  |  |  |  |  |  | I | susceptibility to chikungunya virus infection | Indian | ([53](#_ENREF_53)) |
|  |  |  |  | rs179019 | 0.21 | 12871850 | I2-3 | A | SLE in females | Japanese | ([49](#_ENREF_49)) |
|  |  |  |  | rs179010 | 0.29 | 12884766 | I2-3 | A | SLE in females | Japanese | ([49](#_ENREF_49)) |
|  |  |  |  |  |  |  |  | I | susceptibility to chikungunya virus infection in males | Indian | ([53](#_ENREF_53)) |
|  |  |  |  | rs179009 | 0.18 | 12885361 | I2-3 | I | HCV infection in females | Han Chinese | ([54](#_ENREF_54)) |
|  |  |  |  | rs5741880 | 0.21 | 12869297 | I2-3 | I | susceptibility to chikungunya virus infection in females | Indian | ([53](#_ENREF_53)) |
| X | ENSG00000101916 | ENST00000218032.7 | *TLR8* | rs3764880 | 0.46 | 12906707 | E1 | I | tuberculosis susceptibility in males | Russian | ([55](#_ENREF_55)) |
|  |  |  |  |  |  |  |  | I | tuberculosis susceptibility in males | Han Chinese | ([56](#_ENREF_56)) |
|  |  |  |  |  |  |  |  | I | tuberculosis susceptibility in males | European | ([57](#_ENREF_57)) |
|  |  |  |  | rs2407992 | 0.28 | 12920993 | E2 | A | susceptibility to asthma and related atopic disorders | Danish | ([48](#_ENREF_48)) |
|  |  |  |  | rs3764879 | 0.46 | 12906578 | E1 | A | SLE in females | Danish | ([51](#_ENREF_51)) |
|  |  |  |  |  |  |  |  | I | susceptibility to chikungunya virus infection in males | Indian | ([53](#_ENREF_53)) |
| 3 | ENSG00000239732 | ENST00000360658.2 | *TLR9* | rs5743836 | 0.17 | 52226766 | 5' upstream | I/A | post-infectious irritable bowel syndrome | Caucasian Canadian | ([58](#_ENREF_58)) |
|  |  |  |  |  |  |  |  | A | SLE in females | European Brazilian | ([59](#_ENREF_59)) |
|  |  |  |  |  |  |  |  | I | malaria susceptibility in children | Burundian | ([60](#_ENREF_60)) |
|  |  |  |  |  |  |  |  | N | acute myeloid leukaemia | Polish | ([61](#_ENREF_61)) |
|  |  |  |  | rs352140 | 0.42 | 52222681 | E2 | A | lupus nephritis | Han Chinese | ([62](#_ENREF_62)) |
|  |  |  |  |  |  |  |  | A | ophtalmopathy in male patients with Graves' disease | Taiwanese | ([63](#_ENREF_63)) |
|  |  |  |  |  |  |  |  | I | malaria susceptibility in children | Ghanian | ([64](#_ENREF_64)) |
|  |  |  |  |  |  |  |  | N | cervical cancer | Polish | ([65](#_ENREF_65)) |
|  |  |  |  |  |  |  |  | N | cervical cancer | Han Chinese | ([66](#_ENREF_66)) |
|  |  |  |  |  |  |  |  | A | SLE | Han Chinese | ([67](#_ENREF_67)) |
|  |  |  |  |  |  |  |  | I | CMV infection in infants | Polish | ([68](#_ENREF_68)) |
|  |  |  |  |  |  |  |  | O | neonatal severe hepatitis | Han Chinese | ([32](#_ENREF_32)) |
|  |  |  |  | rs187084 | 0.38 | 52227015 | 5' upstream | N | mortality in prostate cancer patients | Swedish | ([69](#_ENREF_69)) |
|  |  |  |  |  |  |  |  | A | rheumatoid arthritis | Turkish | ([70](#_ENREF_70)) |
|  |  |  |  |  |  |  |  | A | ophtalmopathy in male patients with Graves' disease | Taiwanese | ([63](#_ENREF_63)) |
|  |  |  |  |  |  |  |  | N | cervical cancer | Polish | ([65](#_ENREF_65)) |
|  |  |  |  |  |  |  |  | A | SLE | Taiwanese | ([71](#_ENREF_71)) |
|  |  |  |  |  |  |  |  | N | acute myeloid leukaemia | Polish | ([61](#_ENREF_61)) |
|  |  |  |  |  |  |  |  | I | CMV infection in infants | Polish | ([68](#_ENREF_68)) |
|  |  |  |  |  |  |  |  | O | neonatal severe hepatitis | Han Chinese | ([32](#_ENREF_32)) |
|  |  |  |  |  |  |  |  | O | hip osteoarthitis susceptibility | Han Chinese | ([72](#_ENREF_72)) |
|  |  |  |  | rs352139 | 0.49 | 52224356 | I1-2 | I/A | post-infectious irritable bowel syndrome | Caucasian Canadian | ([58](#_ENREF_58)) |
|  |  |  |  |  |  |  |  | A | lupus nephritis | Han Chinese | ([62](#_ENREF_62)) |
|  |  |  |  |  |  |  |  | I | tuberculosis susceptibility in females | Indonesian | ([73](#_ENREF_73)) |
|  |  |  |  |  |  |  |  | I | malaria susceptibility in children | Ghanian | ([64](#_ENREF_64)) |
|  |  |  |  |  |  |  |  | I | tuberculosis susceptibility | Mexican | ([74](#_ENREF_74)) |
|  |  |  |  |  |  |  |  | I | CMV infection in infants | Polish | ([68](#_ENREF_68)) |
|  |  |  |  |  |  |  |  | O | neonatal severe hepatitis | Han Chinese | ([32](#_ENREF_32)) |
|  |  |  |  |  |  |  |  | I | tuberculosis susceptibility in females | European | ([57](#_ENREF_57)) |
| 4 | ENSG00000137462 | ENST00000260010.6 | *TLR2* | rs1898830 | 0.37 | 153687301 | 5' upstream | I | bacterial vaginosis in HIV-1 infected adolescents | African American | ([75](#_ENREF_75)) |
|  |  |  |  |  |  |  |  | I | congenital CMV infection | Japanese | ([76](#_ENREF_76)) |
|  |  |  |  |  |  |  |  | O | neonatal severe hepatitis | Han Chinese | ([32](#_ENREF_32)) |
|  |  |  |  | rs3804099 | 0.41 | 153703504 | E1 | I | neonatal sepsis | American | ([77](#_ENREF_77)) |
|  |  |  |  |  |  |  |  | N | hepatocellular carcinoma | Chinese | ([78](#_ENREF_78)) |
|  |  |  |  |  |  |  |  | I | bacterial vaginosis in HIV-1 infected | African | ([79](#_ENREF_79)) |
|  |  |  |  |  |  |  |  | A | psoriasis | Chinese | ([80](#_ENREF_80)) |
|  |  |  |  |  |  |  |  | I | tuberculosis | Chinese | ([81](#_ENREF_81)) |
|  |  |  |  |  |  |  |  | I | tuberculosis susceptibility in females | European | ([57](#_ENREF_57)) |
|  |  |  |  | rs3804100 | 0.11 | 153704257 | E1 | A | T1DM, allergic asthma | Norwegian | ([82](#_ENREF_82)) |
|  |  |  |  |  |  |  |  | I | aggressive periodontitis | Japanese | ([83](#_ENREF_83)) |
|  |  |  |  |  |  |  |  | N | hepatocellular carcinoma | Chinese | ([78](#_ENREF_78)) |
|  |  |  |  |  |  |  |  | N | marginal zone lymphoma | American, Australian | ([84](#_ENREF_84)) |
|  |  |  |  |  |  |  |  | I | measles-specific antibody levels following immunization | American | ([85](#_ENREF_85)) |
|  |  |  |  |  |  |  |  | I | congenital CMV infection | Japanese | ([76](#_ENREF_76)) |
|  |  |  |  |  |  |  |  | I | latent tuberculosis infection | Chinese | ([86](#_ENREF_86)) |
|  |  |  |  |  |  |  |  | O | neonatal severe hepatitis | Han Chinese | ([32](#_ENREF_32)) |
|  |  |  |  |  |  |  |  | I | tuberculosis | Chinese | ([81](#_ENREF_81)) |
|  |  |  |  | rs4696480 | 0.42 | 153685974 | 5' upstream | N | oral and laryngeal squamous cell carcinoma | Spanish | ([87](#_ENREF_87)) |
|  |  |  |  |  |  |  |  | A | psoriasis | Turkish | ([88](#_ENREF_88)) |
|  |  |  |  | rs5743704 | 0.01 | 153704799 | E1 | I | recurrent vulvovaginal candidiasis | European | ([89](#_ENREF_89)) |
|  |  |  |  | rs5743708 | 0.01 | 153705165 | E1 | I | Lyme disease susceptibility | European | ([90](#_ENREF_90)) |
|  |  |  |  |  |  |  |  | I | reactive arthritis | Canadian | ([91](#_ENREF_91)) |
|  |  |  |  |  |  |  |  | I | tuberculosis | Chinese | ([86](#_ENREF_86)) |
|  |  |  |  |  |  |  |  | N/I | pneumonia susceptibility in patients with acute myeloid leukaemia | Caucasian | ([92](#_ENREF_92)) |
|  |  |  |  |  |  |  |  | I | nasal Staphylococcus aureus carriage | Polish | ([93](#_ENREF_93)) |
|  |  |  |  | rs7656411 | 0.40 | 153706503 | 3' downstream | N | breast cancer prognosis in women | Korean | ([94](#_ENREF_94)) |
|  |  |  |  | rs7696323 | 0.21 | 153684593 | 5' upstream | I | tuberculosis | Chinese | ([81](#_ENREF_81)) |
|  |  |  |  | rs893629 | 0.04 | 153683816 | 5' upstream | A | arterial thrombosis in patients with SLE | European American and African American | ([95](#_ENREF_95)) |
| 9 | ENSG00000136869 | ENST00000355622.8 | *TLR4* | rs1057317 | 0.36 | 117715764 | E3 | N | hepatocellular carcinoma | Han Chinese | ([96](#_ENREF_96)) |
|  |  |  |  | rs10759932 | 0.18 | 117702866 | 5' upstream | A | acute rejection in kidney transplantation | Korean | ([97](#_ENREF_97)) |
|  |  |  |  |  |  |  |  | N | prostate cancer | American | ([98](#_ENREF_98)) |
|  |  |  |  |  |  |  |  | N | gastric cancer | Chinese | ([99](#_ENREF_99)) |
|  |  |  |  |  |  |  |  | N | gastric precancerous lesions | Han Chinese | ([100](#_ENREF_100)) |
|  |  |  |  | rs10983755 | 0.07 | 117702392 | 5' upstream | N | gastric cancer | Chinese | ([101](#_ENREF_101)) |
|  |  |  |  | rs11536889 | 0.14 | 117715853 | E3 | O | emphysema in smokers | Japanese | ([102](#_ENREF_102)) |
|  |  |  |  |  |  |  |  | I | hepatitis B recurrence after liver transplantation | Han Chinese | ([103](#_ENREF_103)) |
|  |  |  |  |  |  |  |  | N | chemotherapy-induced neutropenia in children with acute lymphoid leukaemia | Dutch | ([104](#_ENREF_104)) |
|  |  |  |  |  |  |  |  | N | prostate cancer | Korean | ([105](#_ENREF_105)) |
|  |  |  |  |  |  |  |  | N | gastric cancer | Chinese | ([99](#_ENREF_99)) |
|  |  |  |  |  |  |  |  | I | sepsis-related organ failure | Caucasian | ([106](#_ENREF_106)) |
|  |  |  |  |  |  |  |  | I | sepsis | Chinese | ([107](#_ENREF_107)) |
|  |  |  |  |  |  |  |  | I | tuberculosis | Chinese | ([81](#_ENREF_81)) |
|  |  |  |  |  |  |  |  | O | intracranial aneurysm | Han Chinese | ([108](#_ENREF_108)) |
|  |  |  |  | rs11536898 | 0.13 | 117717932 | E3 | N | colon cancer | American | ([28](#_ENREF_28)) |
|  |  |  |  | rs12377632 | 0.37 | 117710452 | I2-3 | O | normal tension glaucoma | Japanese | ([109](#_ENREF_109)) |
|  |  |  |  |  |  |  |  | I | tuberculosis | Chinese | ([81](#_ENREF_81)) |
|  |  |  |  | rs1554973 | 0.37 | 117718534 | E3 | O | cervical cytokine concentriations during pregnancy | European American | ([110](#_ENREF_110)) |
|  |  |  |  |  |  |  |  | A | ankylosing spondylitis | Danish | ([111](#_ENREF_111)) |
|  |  |  |  | rs1927907 | 0.18 | 117710486 | I2-3 | O | late-onset Alzheimer's disease | Taiwanese | ([112](#_ENREF_112)) |
|  |  |  |  | rs1927911 | 0.40 | 117707776 | I1-2 | O | myocardial infarction | American | ([113](#_ENREF_113)) |
|  |  |  |  |  |  |  |  | N | prostate cancer | Korean | ([114](#_ENREF_114)) |
|  |  |  |  |  |  |  |  | I | Chlamydia trachomatis infection in women with pelvic inflammatory disease | African American | ([115](#_ENREF_115)) |
|  |  |  |  |  |  |  |  | O | normal tension glaucoma | Japanese | ([109](#_ENREF_109)) |
|  |  |  |  |  |  |  |  | N | chemotherapy-induced neutropenia in children with acute lymphoid leukaemia | Dutch | ([104](#_ENREF_104)) |
|  |  |  |  |  |  |  |  | N | gastric cancer | Chinese | ([116](#_ENREF_116)) |
|  |  |  |  |  |  |  |  | I | pulmonary tuberculosis | Sudanese | ([117](#_ENREF_117)) |
|  |  |  |  |  |  |  |  | N | gastric cancer | Chinese | ([99](#_ENREF_99)) |
|  |  |  |  |  |  |  |  | O | atherosclerotic cerebral infarction | Han Chinese | ([118](#_ENREF_118)) |
|  |  |  |  |  |  |  |  | O | T2DM | Han Chinese | ([119](#_ENREF_119)) |
|  |  |  |  | rs1927914 | 0.49 | 117702447 | 5' upstream | O | normal tension glaucoma | Japanese | ([109](#_ENREF_109)) |
|  |  |  |  |  |  |  |  | O | Parkinson's disease | Han Chinese | ([120](#_ENREF_120)) |
|  |  |  |  |  |  |  |  | O | T2DM | Chinese | ([119](#_ENREF_119)) |
|  |  |  |  |  |  |  |  | O | Parkinson's disease | Han Chinese | ([121](#_ENREF_121)) |
|  |  |  |  | rs2149356 | 0.48 | 117711921 | I2-3 | O | normal tension glaucoma | Japanese | ([109](#_ENREF_109)) |
|  |  |  |  |  |  |  |  | O | gout | Han Chinese | ([122](#_ENREF_122)) |
|  |  |  |  |  |  |  |  | O | glaucoma | Japanese | ([123](#_ENREF_123)) |
|  |  |  |  |  |  |  |  | N | gastric cancer | Chinese | ([99](#_ENREF_99)) |
|  |  |  |  |  |  |  |  | O | gout | European and Polynesian | ([124](#_ENREF_124)) |
|  |  |  |  |  |  |  |  | O | neonatal severe hepatitis | Han Chinese | ([32](#_ENREF_32)) |
|  |  |  |  |  |  |  |  | O | primary open angle glaucoma | Chinese | ([125](#_ENREF_125)) |
|  |  |  |  | rs41426344 | 0.01 | 117715655 | E3 | A | rheumatoid arthritis | Han Chinese | ([126](#_ENREF_126)) |
|  |  |  |  | rs4986790 | 0.06 | 117713024 | E3 | O | diabetic neuropathy in patients with T2DM | Caucasian | ([127](#_ENREF_127)) |
|  |  |  |  |  |  |  |  | O | acute coronary syndrome | European | ([128](#_ENREF_128)) |
|  |  |  |  |  |  |  |  | O | carotid atherosclerosis | Italian | ([129](#_ENREF_129)) |
|  |  |  |  |  |  |  |  | I | Gram-negative bacterial infection in patients with systemic inflammatory response syndrome | American | ([130](#_ENREF_130)) |
|  |  |  |  |  |  |  |  | I | severe respiratory syncytial virus disease | Jewish | ([131](#_ENREF_131)) |
|  |  |  |  |  |  |  |  | A | acute rejection after lung transplantation | American | ([132](#_ENREF_132)) |
|  |  |  |  |  |  |  |  | A | giant cell arteritis | Spanish | ([133](#_ENREF_133)) |
|  |  |  |  |  |  |  |  | I | clinical malaria | Brazilian Amazonian | ([134](#_ENREF_134)) |
|  |  |  |  |  |  |  |  | I | IL-4 secretion after measles vaccination | American | ([135](#_ENREF_135)) |
|  |  |  |  |  |  |  |  | A | early-onset preeclampsia, HELLP syndrome | Dutch | ([136](#_ENREF_136)) |
|  |  |  |  |  |  |  |  | I | recurrent cystitis in women | Caucasian | ([137](#_ENREF_137)) |
|  |  |  |  |  |  |  |  | I | prognosis of chronic HBV infection in males | Caucasian | ([138](#_ENREF_138)) |
|  |  |  |  |  |  |  |  | N | gastric cancer | Brazilian | ([139](#_ENREF_139)) |
|  |  |  |  |  |  |  |  | I | bacterial vaginosis in HIV-1 infected adolescents | African American | ([75](#_ENREF_75)) |
|  |  |  |  |  |  |  |  | I | Gram-negative bacterial infection in very low birth weight infants | American | ([140](#_ENREF_140)) |
|  |  |  |  |  |  |  |  | I | HBsAg seroclearance and seroconversion in HBV-infected patients | Taiwanese | ([141](#_ENREF_141)) |
|  |  |  |  |  |  |  |  | I | Mycobacterium avium subspecies paratuberculosis-positivity in paediatric Crohn's disease patients | Australian | ([142](#_ENREF_142)) |
|  |  |  |  |  |  |  |  | I | hepatitis C virus infection | Saudi Arabian | ([143](#_ENREF_143)) |
|  |  |  |  |  |  |  |  | I | chronic cavitary pulmonary aspergillosis | European | ([144](#_ENREF_144)) |
|  |  |  |  |  |  |  |  | N | non-Hodgkin lymphoma | Han Chinese | ([145](#_ENREF_145)) |
|  |  |  |  | rs5030717 | 0.10 | 117711556 | I2-3 | I | childhood otitis media | Finnish | ([146](#_ENREF_146)) |
|  |  |  |  | rs7873784 | 0.14 | 117716658 | E3 | N | colorectal cancer | American | ([147](#_ENREF_147)) |
|  |  |  |  |  |  |  |  | A | rheumatoid arthritis | Han Chinese | ([126](#_ENREF_126)) |
|  |  |  |  |  |  |  |  | I | tuberculosis | Chinese | ([86](#_ENREF_86)) |
|  |  |  |  |  |  |  |  | O | Parkinson's disease | Han Chinese | ([121](#_ENREF_121)) |
| 3 | ENSG00000172936 | ENST00000650905.2 | *MYD88* | rs7744 | 0.14 | 38142530 | E5 | A | Buerger disease | Japanese | ([148](#_ENREF_148)) |
|  |  |  |  |  |  |  |  | A | treatment response in patients with rheumatoid arthritis | British | ([149](#_ENREF_149)) |
|  |  |  |  | rs6853 | 0.13 | 38142879 | E5 | I | pulmonary tuberculosis | Italian | ([150](#_ENREF_150)) |
|  |  |  |  | rs4988453 | 0.04 | 38137763 | 5' upstream | I | tuberculosis susceptibility in females | European | ([57](#_ENREF_57)) |
| 19 | ENSG00000127666 | ENST00000248244.5 | ***TRIF/TICAM1*** | rs8120 | 0.31 | 4816148 | E2 | N | follicular and papillary thyroid cancer | German | ([151](#_ENREF_151)) |
|  |  |  |  | rs11466711 | 0.19 | 4831201 | I1-2 | I | community acquired pneumonia in children | Chinese | ([152](#_ENREF_152)) |
| 4 | ENSG00000109320 | ENST00000226574.9 | *NFKB1* | rs11940017 | 0.14 | 102499602 | 5' upstream | O | ischemic stroke | Korean | ([153](#_ENREF_153)) |
|  |  |  |  | rs1585215 | 0.25 | 102523317 | I1-2 | N | Hodgkin lymphoma | American | ([154](#_ENREF_154)) |
|  |  |  |  |  |  |  |  | N | non-Hodgkin lymphoma | Han Chinese | ([145](#_ENREF_145)) |
|  |  |  |  | rs230496 | 0.41 | 102567334 | I6-7 | N | liver cancer | Chinese | ([155](#_ENREF_155)) |
|  |  |  |  | rs230529 | 0.41 | 102536261 | I4-5 | O | treatment refractory scizophrenia | Han Chinese | ([156](#_ENREF_156)) |
|  |  |  |  | rs28362491 | 0.42 | 102500998 | 5' upstream | O | coronary artery disease in females | Han and Uygur Chinese | ([157](#_ENREF_157)) |
|  |  |  |  |  |  |  |  | N | cervical squamous cell carcinoma | Han Chinese | ([158](#_ENREF_158)) |
|  |  |  |  |  |  |  |  | N | colorectal cancer | Danish | ([159](#_ENREF_159)) |
|  |  |  |  |  |  |  |  | A | SLE | Chinese | ([160](#_ENREF_160)) |
|  |  |  |  |  |  |  |  | I/A | dilated cardiomyopathy | Han Chinese | ([161](#_ENREF_161)) |
|  |  |  |  |  |  |  |  | N | colorectal cancer | Malaysian | ([162](#_ENREF_162)) |
|  |  |  |  |  |  |  |  | N | liver cancer | Chinese | ([155](#_ENREF_155)) |
|  |  |  |  |  |  |  |  | N | papillary thyroid cancer | Chinese | ([163](#_ENREF_163)) |
|  |  |  |  |  |  |  |  | A | Behcet's disease | Turkish | ([164](#_ENREF_164)) |
|  |  |  |  |  |  |  |  | O | coronary artery disease | Uygur Chinese | ([165](#_ENREF_165)) |
|  |  |  |  |  |  |  |  | O | coronary artery disease | Han Chinese | ([166](#_ENREF_166)) |
|  |  |  |  |  |  |  |  | O | coronary artery disease | Han Chinese | ([167](#_ENREF_167)) |
|  |  |  |  |  |  |  |  | I | HCV infection | Han Chinese | ([168](#_ENREF_168)) |
|  |  |  |  |  |  |  |  | O | acute coronary syndrome | Han Chinese | ([169](#_ENREF_169)) |
|  |  |  |  |  |  |  |  | N | breast cancer | Thai | ([170](#_ENREF_170)) |
|  |  |  |  | rs3774937 | 0.25 | 102513096 | I1-2 | A | acute rejection after renal transplantation | Hispanic American | ([171](#_ENREF_171)) |
|  |  |  |  | rs4648022 | 0.04 | 102575280 | I6-7 | N | non-Hodgkin lymphoma | American | ([172](#_ENREF_172)) |
|  |  |  |  | rs4648127 | 0.04 | 102614748 | I23-24 | N | lung cancer | American | ([173](#_ENREF_173)) |
|  |  |  |  | rs4699030 | 0.42 | 102582667 | I9-10 | O | treatment refractory scizophrenia | Han Chinese | ([156](#_ENREF_156)) |
| 10 | ENSG00000077150 | ENST00000428099.6 | *NFKB2* | rs11574851 | 0.03 | 102401202 | E19 | A | rheumatoid arthritis susceptibility among anti-citrullinated protein antibodies-positive patients | European | ([174](#_ENREF_174)) |
| 11 | ENSG00000173039 | ENST00000406246.8 | *RELA* | rs1049728 | 0.03 | 65653646 | E11 | O | soluble ICAM-1 concentration in healthy women | American | ([175](#_ENREF_175)) |
| 2 | ENSG00000162924 | ENST00000394479.4 | *REL* | rs13017599 | 0.13 | 60937196 | 3' downstream | A | psoriatic arthritis | British and Irish | ([176](#_ENREF_176)) |
|  |  |  |  |  |  |  |  | A | rheumatoid arthritis | American and Canadian | ([177](#_ENREF_177)) |
|  |  |  |  | rs13031237 | 0.13 | 60908994 | I4-5 | A | rheumatoid arthritis | American and Canadian | ([177](#_ENREF_177)) |
|  |  |  |  |  |  |  |  | A | rheumatoid arthritis | British | ([178](#_ENREF_178)) |
|  |  |  |  |  |  |  |  | A | early-onset psoriasis | British | ([179](#_ENREF_179)) |
|  |  |  |  | rs842647 | 0.45 | 60892336 | I2-3 | A | coeliac disease | European | ([180](#_ENREF_180)) |
| 7 | ENSG00000136244 | ENST00000404625.5 | *IL6* | rs1474347 | 0.17 | 22728505 | I3-4 | A | rheumatoid arthritis | Han Chinese | ([181](#_ENREF_181)) |
|  |  |  |  | rs1524107 | 0.31 | 22728600 | I3-4 | N | endometrial cancer | Han Chinese | ([182](#_ENREF_182)) |
|  |  |  |  |  |  |  |  | N | papillary thyroid cancer | Chinese | ([183](#_ENREF_183)) |
|  |  |  |  | rs1800795 | 0.14 | 22727026 | I1-2 | O | T2DM | Caucasian | ([184](#_ENREF_184)) |
|  |  |  |  |  |  |  |  | A | new onset diabetes after renal transplantation | European | ([185](#_ENREF_185)) |
|  |  |  |  |  |  |  |  | I | bronchiolitis obliterans after lung transplantation | American | ([186](#_ENREF_186)) |
|  |  |  |  |  |  |  |  | I/N | Kaposi sarcoma in HIV infected males | American | ([187](#_ENREF_187)) |
|  |  |  |  |  |  |  |  | I/A | post-infectious irritable bowel syndrome | Caucasian Canadian | ([58](#_ENREF_58)) |
|  |  |  |  |  |  |  |  | O | mortality after acute coronary syndrome in males | Italian | ([188](#_ENREF_188)) |
|  |  |  |  |  |  |  |  | O | hypertension | Taiwanese | ([189](#_ENREF_189)) |
|  |  |  |  |  |  |  |  | A | long-term renal allograft survival | Caucasian European | ([190](#_ENREF_190)) |
|  |  |  |  |  |  |  |  | O | stroke after cardiac surgery | American | ([191](#_ENREF_191)) |
|  |  |  |  |  |  |  |  | O | Alzheimer's disease | Italian | ([192](#_ENREF_192)) |
|  |  |  |  |  |  |  |  | O | longevity | Finnish | ([193](#_ENREF_193)) |
|  |  |  |  | rs2066992 | 0.31 | 22728630 | I3-4 | N | endometrial cancer | Han Chinese | ([182](#_ENREF_182)) |
|  |  |  |  |  |  |  |  | N | papillary thyroid cancer | Chinese | ([183](#_ENREF_183)) |
|  |  |  |  | rs2069830 | 0.03 | 22727518 | E3 | O | stroke in young, non-smoking women | American | ([194](#_ENREF_194)) |
|  |  |  |  | rs2069832 | 0.14 | 22727814 | I3-4 | O | stroke in young women | African American | ([194](#_ENREF_194)) |
|  |  |  |  |  |  |  |  | I | acute lung injury in patients with systemic inflammatory response syndrome | Caucasian American | ([195](#_ENREF_195)) |
|  |  |  |  | rs2069837 | 0.12 | 22728408 | I3-4 | N | cervical cancer | Chinese | ([196](#_ENREF_196)) |
|  |  |  |  |  |  |  |  | I | pulmonary tuberculosis | Tibetan Chinese | ([197](#_ENREF_197)) |
|  |  |  |  | rs2069845 | 0.25 | 22730530 | I5-6 | O | obesity in children | Indian | ([198](#_ENREF_198)) |
| 4 | ENSG00000169429 | ENST00000307407.8 | *CXCL8* | rs4073 | 0.48 | 73740307 | 5' upstream | I | acne vulgaris | Pakistani | ([199](#_ENREF_199)) |
|  |  |  |  |  |  |  |  | O | myocardial infarction in males | Swedish | ([200](#_ENREF_200)) |
|  |  |  |  |  |  |  |  | N | non small cell lung cancer | Tunisian | ([201](#_ENREF_201)) |
|  |  |  |  |  |  |  |  | O | idiopathic pulmonary fibrosis | Korean | ([202](#_ENREF_202)) |
|  |  |  |  |  |  |  |  | A | childhood IgA nephropathy | Korean | ([203](#_ENREF_203)) |
|  |  |  |  |  |  |  |  | A | erosive oral lichen planus | Chinese | ([204](#_ENREF_204)) |
|  |  |  |  |  |  |  |  | N | survival in patients with follicular lymphoma | American | ([205](#_ENREF_205)) |
|  |  |  |  |  |  |  |  | A | childhood asthma | Tunisian | ([206](#_ENREF_206)) |
|  |  |  |  |  |  |  |  | N | survival in patients with gastric cancer | Chilean | ([207](#_ENREF_207)) |
|  |  |  |  | rs2227306 | 0.26 | 73741338 | I1-2 | A | Graves' disease | Chinese | ([208](#_ENREF_208)) |
|  |  |  |  |  |  |  |  | A | childhood IgA nephropathy | Korean | ([203](#_ENREF_203)) |
|  |  |  |  |  |  |  |  | A | childhood asthma | Tunisian | ([206](#_ENREF_206)) |
|  |  |  |  | rs2227307 | 0.42 | 73740952 | I1-2 | I | chronic periodontitis | Brazilian | ([209](#_ENREF_209)) |
|  |  |  |  |  |  |  |  | O | idiopathic pulmonary fibrosis | Korean | ([202](#_ENREF_202)) |
|  |  |  |  |  |  |  |  | I | invasive aspergillosis among immunocompromised patients | European | ([210](#_ENREF_210)) |
|  |  |  |  | rs2227532 | 0.03 | 73739815 | 5' upstream | N | gastric cancer | Brazilian | ([139](#_ENREF_139)) |

**References**

1. Ovsyannikova IG, Haralambieva IH, Dhiman N, O'Byrne MM, Pankratz VS, Jacobson RM, et al. Polymorphisms in the vitamin A receptor and innate immunity genes influence the antibody response to rubella vaccination. J Infect Dis. 2010;201(2):207-13.

2. Biggins SW, Trotter J, Gralla J, Burton JR, Jr., Bambha KM, Dodge J, et al. Differential effects of donor and recipient IL28B and DDX58 SNPs on severity of HCV after liver transplantation. Journal of hepatology. 2013;58(5):969-76.

3. Ovsyannikova IG, Salk HM, Larrabee BR, Pankratz VS, Poland GA. Single-nucleotide polymorphism associations in common with immune responses to measles and rubella vaccines. Immunogenetics. 2014;66(11):663-9.

4. Li YP, Li M, Jia XL, Deng HL, Wang WJ, Wu FP, et al. Association of gene polymorphisms of pattern-recognition receptor signaling pathway with the risk and severity of hand, foot, and mouth disease caused by enterovirus 71 in Chinese Han population. J Med Virol. 2018;90(4):692-8.

5. Haralambieva IH, Ovsyannikova IG, Umlauf BJ, Vierkant RA, Shane Pankratz V, Jacobson RM, et al. Genetic polymorphisms in host antiviral genes: associations with humoral and cellular immunity to measles vaccine. Vaccine. 2011;29(48):8988-97.

6. Moumad K, Lascorz J, Bevier M, Khyatti M, Ennaji MM, Benider A, et al. Genetic polymorphisms in host innate immune sensor genes and the risk of nasopharyngeal carcinoma in North Africa. G3 (Bethesda). 2013;3(6):971-7.

7. Li Y, Liao W, Cargill M, Chang M, Matsunami N, Feng BJ, et al. Carriers of rare missense variants in IFIH1 are protected from psoriasis. J Invest Dermatol. 2010;130(12):2768-72.

8. Molineros JE, Maiti AK, Sun C, Looger LL, Han S, Kim-Howard X, et al. Admixture mapping in lupus identifies multiple functional variants within IFIH1 associated with apoptosis, inflammation, and autoantibody production. PLoS genetics. 2013;9(2):e1003222.

9. Jermendy A, Szatmari I, Laine AP, Lukacs K, Horvath KH, Korner A, et al. The interferon-induced helicase IFIH1 Ala946Thr polymorphism is associated with type 1 diabetes in both the high-incidence Finnish and the medium-incidence Hungarian populations. Diabetologia. 2010;53(1):98-102.

10. Smyth DJ, Cooper JD, Bailey R, Field S, Burren O, Smink LJ, et al. A genome-wide association study of nonsynonymous SNPs identifies a type 1 diabetes locus in the interferon-induced helicase (IFIH1) region. Nature genetics. 2006;38(6):617-9.

11. Emdin CA, Khera AV, Chaffin M, Klarin D, Natarajan P, Aragam K, et al. Analysis of predicted loss-of-function variants in UK Biobank identifies variants protective for disease. Nature communications. 2018;9(1):1613.

12. Nejentsev S, Walker N, Riches D, Egholm M, Todd JA. Rare variants of IFIH1, a gene implicated in antiviral responses, protect against type 1 diabetes. Science. 2009;324(5925):387-9.

13. Chistiakov DA, Voronova NV, Savost'Anov KV, Turakulov RI. Loss-of-function mutations E6 27X and I923V of IFIH1 are associated with lower poly(I:C)-induced interferon-beta production in peripheral blood mononuclear cells of type 1 diabetes patients. Hum Immunol. 2010;71(11):1128-34.

14. Enevold C, Oturai AB, Sorensen PS, Ryder LP, Koch-Henriksen N, Bendtzen K. Multiple sclerosis and polymorphisms of innate pattern recognition receptors TLR1-10, NOD1-2, DDX58, and IFIH1. J Neuroimmunol. 2009;212(1-2):125-31.

15. Yang H, Wang Z, Xu K, Gu R, Chen H, Yu D, et al. IFIH1 gene polymorphisms in type 1 diabetes: genetic association analysis and genotype-phenotype correlation in Chinese Han population. Autoimmunity. 2012;45(3):226-32.

16. Wang C, Ahlford A, Laxman N, Nordmark G, Eloranta ML, Gunnarsson I, et al. Contribution of IKBKE and IFIH1 gene variants to SLE susceptibility. Genes Immun. 2013;14(4):217-22.

17. Liu J, Tang LY, Wang YG, Lu SY, Zhang EN, Wang ZG, et al. Identification of MAVS as a Novel Risk Factor for the Development of Osteoarthritis. Aging Dis. 2018;9(1):40-50.

18. Pothlichet J, Niewold TB, Vitour D, Solhonne B, Crow MK, Si-Tahar M. A loss-of-function variant of the antiviral molecule MAVS is associated with a subset of systemic lupus patients. EMBO Mol Med. 2011;3(3):142-52.

19. Slattery ML, Lundgreen A, Bondurant KL, Wolff RK. Interferon-signaling pathway: associations with colon and rectal cancer risk and subsequent survival. Carcinogenesis. 2011;32(11):1660-7.

20. Santana-de Anda K, Gomez-Martin D, Monsivais-Urenda AE, Salgado-Bustamante M, Gonzalez-Amaro R, Alcocer-Varela J. Interferon regulatory factor 3 as key element of the interferon signature in plasmacytoid dendritic cells from systemic lupus erythematosus patients: novel genetic associations in the Mexican mestizo population. Clin Exp Immunol. 2014;178(3):428-37.

21. Lin LH, Ling P, Liu MF. The potential role of interferon-regulatory factor 7 among Taiwanese patients with systemic lupus erythematosus. J Rheumatol. 2011;38(9):1914-9.

22. Fu Q, Zhao J, Qian X, Wong JL, Kaufman KM, Yu CY, et al. Association of a functional IRF7 variant with systemic lupus erythematosus. Arthritis Rheum. 2011;63(3):749-54.

23. Carmona FD, Gutala R, Simeon CP, Carreira P, Ortego-Centeno N, Vicente-Rabaneda E, et al. Novel identification of the IRF7 region as an anticentromere autoantibody propensity locus in systemic sclerosis. Annals of the rheumatic diseases. 2012;71(1):114-9.

24. Chuang SC, Hsi E, Wang SN, Yu ML, Lee KT, Juo SH. Polymorphism at the mucin-like protocadherin gene influences susceptibility to gallstone disease. Clinica chimica acta; international journal of clinical chemistry. 2011;412(23-24):2089-93.

25. Paradowska-Gorycka A, Wajda A, Stypinska B, Walczuk E, Walczyk M, Felis-Giemza A, et al. Interferons (IFN-A/-B/-G) Genetic Variants in Patients with Mixed Connective Tissue Disease (MCTD). J Clin Med. 2019;8(12).

26. Fujita M, Scheurer ME, Decker SA, McDonald HA, Kohanbash G, Kastenhuber ER, et al. Role of type 1 IFNs in antiglioma immunosurveillance--using mouse studies to guide examination of novel prognostic markers in humans. Clinical cancer research : an official journal of the American Association for Cancer Research. 2010;16(13):3409-19.

27. Yeyeodu ST, Kidd LR, Oprea-Ilies GM, Burns BG, Vancleave TT, Shim JY, et al. IRAK4 and TLR3 Sequence Variants may Alter Breast Cancer Risk among African-American Women. Front Immunol. 2013;4:338.

28. Slattery ML, Herrick JS, Bondurant KL, Wolff RK. Toll-like receptor genes and their association with colon and rectal cancer development and prognosis. International journal of cancer Journal international du cancer. 2012;130(12):2974-80.

29. Svensson A, Tunback P, Nordstrom I, Padyukov L, Eriksson K. Polymorphisms in Toll-like receptor 3 confer natural resistance to human herpes simplex virus type 2 infection. J Gen Virol. 2012;93(Pt 8):1717-24.

30. Qian F, Bolen CR, Jing C, Wang X, Zheng W, Zhao H, et al. Impaired toll-like receptor 3-mediated immune responses from macrophages of patients chronically infected with hepatitis C virus. Clin Vaccine Immunol. 2013;20(2):146-55.

31. Al-Qahtani A, Al-Ahdal M, Abdo A, Sanai F, Al-Anazi M, Khalaf N, et al. Toll-like receptor 3 polymorphism and its association with hepatitis B virus infection in Saudi Arabian patients. J Med Virol. 2012;84(9):1353-9.

32. Qiu X, Dong Y, Cao Y, Luo Y. Correlation between TLR2, TLR3, TLR4, and TLR9 polymorphisms and susceptibility to and prognosis of severe hepatitis among the newborns. Journal of clinical laboratory analysis. 2018;32(3).

33. Huang X, Li H, Wang J, Huang C, Lu Y, Qin X, et al. Genetic polymorphisms in Toll-like receptor 3 gene are associated with the risk of hepatitis B virus-related liver diseases in a Chinese population. Gene. 2015;569(2):218-24.

34. Wang J, Liu Y, Liu Y, Zhu K, Xie A. The association between TLR3 rs3775290 polymorphism and sporadic Parkinson's disease in Chinese Han population. Neuroscience letters. 2020;728:135005.

35. Yang Z, Stratton C, Francis PJ, Kleinman ME, Tan PL, Gibbs D, et al. Toll-like receptor 3 and geographic atrophy in age-related macular degeneration. The New England journal of medicine. 2008;359(14):1456-63.

36. Castro FA, Forsti A, Buch S, Kalthoff H, Krauss C, Bauer M, et al. TLR-3 polymorphism is an independent prognostic marker for stage II colorectal cancer. European journal of cancer. 2011;47(8):1203-10.

37. Sironi M, Biasin M, Cagliani R, Forni D, De Luca M, Saulle I, et al. A common polymorphism in TLR3 confers natural resistance to HIV-1 infection. J Immunol. 2012;188(2):818-23.

38. Moore CE, Hennig BJ, Perrett KP, Hoe JC, Lee SJ, Fletcher H, et al. Single nucleotide polymorphisms in the Toll-like receptor 3 and CD44 genes are associated with persistence of vaccine-induced immunity to the serogroup C meningococcal conjugate vaccine. Clin Vaccine Immunol. 2012;19(3):295-303.

39. Ishizaki Y, Takemoto M, Kira R, Kusuhara K, Torisu H, Sakai Y, et al. Association of toll-like receptor 3 gene polymorphism with subacute sclerosing panencephalitis. Journal of neurovirology. 2008;14(6):486-91.

40. Huik K, Avi R, Pauskar M, Kallas E, Jogeda EL, Karki T, et al. Association between TLR3 rs3775291 and resistance to HIV among highly exposed Caucasian intravenous drug users. Infect Genet Evol. 2013;20:78-82.

41. Laska MJ, Troldborg A, Hansen B, Stengaard-Pedersen K, Junker P, Nexo BA, et al. Polymorphisms within Toll-like receptors are associated with systemic lupus erythematosus in a cohort of Danish females. Rheumatology (Oxford). 2014;53(1):48-55.

42. Laska MJ, Hansen B, Troldborg A, Lorenzen T, Stengaard-Pedersen K, Junker P, et al. A non-synonymous single-nucleotide polymorphism in the gene encoding Toll-like Receptor 3 (TLR3) is associated with sero-negative rheumatoid arthritis (RA) in a Danish population. BMC research notes. 2014;7:716.

43. Ikezoe K, Handa T, Tanizawa K, Kubo T, Ito I, Sokai A, et al. A toll-like receptor 3 single nucleotide polymorphism in Japanese patients with sarcoidosis. Tissue Antigens. 2015;85(3):204-8.

44. Fan L, Zhou P, Chen AX, Liu GY, Yu KD, Shao ZM. Toll-like receptor 3 -926T>A increased the risk of breast cancer through decreased transcriptional activity. Oncoimmunology. 2019;8(12):e1673126.

45. Park JY, Amankwah EK, Anic GM, Lin HY, Walls B, Park H, et al. Gene variants in angiogenesis and lymphangiogenesis and cutaneous melanoma progression. Cancer epidemiology, biomarkers & prevention : a publication of the American Association for Cancer Research, cosponsored by the American Society of Preventive Oncology. 2013;22(5):827-34.

46. Al-Anazi MR, Matou-Nasri S, Abdo AA, Sanai FM, Alkahtani S, Alarifi S, et al. Association of Toll-Like Receptor 3 Single-Nucleotide Polymorphisms and Hepatitis C Virus Infection. J Immunol Res. 2017;2017:1590653.

47. Edwards AO, Chen D, Fridley BL, James KM, Wu Y, Abecasis G, et al. Toll-like receptor polymorphisms and age-related macular degeneration. Investigative ophthalmology & visual science. 2008;49(4):1652-9.

48. Moller-Larsen S, Nyegaard M, Haagerup A, Vestbo J, Kruse TA, Borglum AD. Association analysis identifies TLR7 and TLR8 as novel risk genes in asthma and related disorders. Thorax. 2008;63(12):1064-9.

49. Kawasaki A, Furukawa H, Kondo Y, Ito S, Hayashi T, Kusaoi M, et al. TLR7 single-nucleotide polymorphisms in the 3' untranslated region and intron 2 independently contribute to systemic lupus erythematosus in Japanese women: a case-control association study. Arthritis Res Ther. 2011;13(2):R41.

50. Shen N, Fu Q, Deng Y, Qian X, Zhao J, Kaufman KM, et al. Sex-specific association of X-linked Toll-like receptor 7 (TLR7) with male systemic lupus erythematosus. Proceedings of the National Academy of Sciences of the United States of America. 2010;107(36):15838-43.

51. Enevold C, Nielsen CH, Jacobsen RS, Hermansen ML, Molbo D, Avlund K, et al. Single nucleotide polymorphisms in genes encoding toll-like receptors 7, 8 and 9 in Danish patients with systemic lupus erythematosus. Molecular biology reports. 2014;41(9):5755-63.

52. Raafat, II, El Guindy N, Shahin RMH, Samy LA, El Refai RM. Toll-like receptor 7 gene single nucleotide polymorphisms and the risk for systemic lupus erythematosus: a case-control study. Z Rheumatol. 2018;77(5):416-20.

53. Dutta SK, Tripathi A. Association of toll-like receptor polymorphisms with susceptibility to chikungunya virus infection. Virology. 2017;511:207-13.

54. Yue M, Gao CF, Wang JJ, Wang CJ, Feng L, Wang J, et al. Toll-like receptor 7 variations are associated with the susceptibility to HCV infection among Chinese females. Infect Genet Evol. 2014;27:264-70.

55. Davila S, Hibberd ML, Hari Dass R, Wong HE, Sahiratmadja E, Bonnard C, et al. Genetic association and expression studies indicate a role of toll-like receptor 8 in pulmonary tuberculosis. PLoS genetics. 2008;4(10):e1000218.

56. Wang MG, Zhang MM, Wang Y, Wu SQ, Zhang M, He JQ. Association of TLR8 and TLR9 polymorphisms with tuberculosis in a Chinese Han population: a case-control study. BMC Infect Dis. 2018;18(1):561.

57. Varzari A, Deyneko IV, Vladei I, Grallert H, Schieck M, Tudor E, et al. Genetic variation in TLR pathway and the risk of pulmonary tuberculosis in a Moldavian population. Infect Genet Evol. 2019;68:84-90.

58. Villani AC, Lemire M, Thabane M, Belisle A, Geneau G, Garg AX, et al. Genetic risk factors for post-infectious irritable bowel syndrome following a waterborne outbreak of gastroenteritis. Gastroenterology. 2010;138(4):1502-13.

59. dos Santos BP, Valverde JV, Rohr P, Monticielo OA, Brenol JC, Xavier RM, et al. TLR7/8/9 polymorphisms and their associations in systemic lupus erythematosus patients from southern Brazil. Lupus. 2012;21(3):302-9.

60. Esposito S, Molteni CG, Zampiero A, Baggi E, Lavizzari A, Semino M, et al. Role of polymorphisms of toll-like receptor (TLR) 4, TLR9, toll-interleukin 1 receptor domain containing adaptor protein (TIRAP) and FCGR2A genes in malaria susceptibility and severity in Burundian children. Malar J. 2012;11:196.

61. Rybka J, Gebura K, Wrobel T, Wysoczanska B, Stefanko E, Kuliczkowski K, et al. Variations in genes involved in regulation of the nuclear factor - kappaB pathway and the risk of acute myeloid leukaemia. Int J Immunogenet. 2016;43(2):101-6.

62. Zhou XJ, Lv JC, Cheng WR, Yu L, Zhao MH, Zhang H. Association of TLR9 gene polymorphisms with lupus nephritis in a Chinese Han population. Clin Exp Rheumatol. 2010;28(3):397-400.

63. Liao WL, Chen RH, Lin HJ, Liu YH, Chen WC, Tsai Y, et al. Toll-like receptor gene polymorphisms are associated with susceptibility to Graves' ophthalmopathy in Taiwan males. BMC Med Genet. 2010;11:154.

64. Omar AH, Yasunami M, Yamazaki A, Shibata H, Ofori MF, Akanmori BD, et al. Toll-like receptor 9 (TLR9) polymorphism associated with symptomatic malaria: a cohort study. Malar J. 2012;11:168.

65. Roszak A, Lianeri M, Sowinska A, Jagodzinski PP. Involvement of Toll-like Receptor 9 polymorphism in cervical cancer development. Molecular biology reports. 2012;39(8):8425-30.

66. Lai ZZ, Ni Z, Pan XL, Song L. Toll-like receptor 9 (TLR9) gene polymorphisms associated with increased susceptibility of human papillomavirus-16 infection in patients with cervical cancer. J Int Med Res. 2013;41(4):1027-36.

67. Zhang J, Zhu Q, Meng F, Lei H, Zhao Y. Association study of TLR-9 polymorphisms and systemic lupus erythematosus in northern Chinese Han population. Gene. 2014;533(1):385-8.

68. Paradowska E, Jablonska A, Studzinska M, Skowronska K, Suski P, Wisniewska-Ligier M, et al. TLR9 -1486T/C and 2848C/T SNPs Are Associated with Human Cytomegalovirus Infection in Infants. PloS one. 2016;11(4):e0154100.

69. Stark JR, Wiklund F, Gronberg H, Schumacher F, Sinnott JA, Stampfer MJ, et al. Toll-like receptor signaling pathway variants and prostate cancer mortality. Cancer epidemiology, biomarkers & prevention : a publication of the American Association for Cancer Research, cosponsored by the American Society of Preventive Oncology. 2009;18(6):1859-63.

70. Etem EO, Elyas H, Ozgocmen S, Yildirim A, Godekmerdan A. The investigation of toll-like receptor 3, 9 and 10 gene polymorphisms in Turkish rheumatoid arthritis patients. Rheumatol Int. 2011;31(10):1369-74.

71. Huang CM, Huang PH, Chen CL, Lin YJ, Tsai CH, Huang WL, et al. Association of toll-like receptor 9 gene polymorphism in Chinese patients with systemic lupus erythematosus in Taiwan. Rheumatol Int. 2012;32(7):2105-9.

72. Yi X, Xu E, Xiao Y, Cai X. Evaluation of the Relationship Between Common Variants in the TLR-9 Gene and Hip Osteoarthritis Susceptibility. Genetic testing and molecular biomarkers. 2019;23(6):373-9.

73. Kobayashi K, Yuliwulandari R, Yanai H, Naka I, Lien LT, Hang NT, et al. Association of TLR polymorphisms with development of tuberculosis in Indonesian females. Tissue Antigens. 2012;79(3):190-7.

74. Torres-Garcia D, Cruz-Lagunas A, Garcia-Sancho Figueroa MC, Fernandez-Plata R, Baez-Saldana R, Mendoza-Milla C, et al. Variants in toll-like receptor 9 gene influence susceptibility to tuberculosis in a Mexican population. J Transl Med. 2013;11:220.

75. Royse KE, Kempf MC, McGwin G, Jr., Wilson CM, Tang J, Shrestha S. Toll-like receptor gene variants associated with bacterial vaginosis among HIV-1 infected adolescents. J Reprod Immunol. 2012;96(1-2):84-9.

76. Taniguchi R, Koyano S, Suzutani T, Goishi K, Ito Y, Morioka I, et al. Polymorphisms in TLR-2 are associated with congenital cytomegalovirus (CMV) infection but not with congenital CMV disease. Int J Infect Dis. 2013;17(12):e1092-7.

77. Abu-Maziad A, Schaa K, Bell EF, Dagle JM, Cooper M, Marazita ML, et al. Role of polymorphic variants as genetic modulators of infection in neonatal sepsis. Pediatr Res. 2010;68(4):323-9.

78. Junjie X, Songyao J, Minmin S, Yanyan S, Baiyong S, Xiaxing D, et al. The association between Toll-like receptor 2 single-nucleotide polymorphisms and hepatocellular carcinoma susceptibility. BMC cancer. 2012;12:57.

79. Mackelprang RD, Scoville CW, Cohen CR, Ondondo RO, Bigham AW, Celum C, et al. Toll-like receptor gene variants and bacterial vaginosis among HIV-1 infected and uninfected African women. Genes Immun. 2015;16(5):362-5.

80. Shi G, Wang T, Li S, Cheng Y, Sheng P, Fan Y, et al. TLR2 and TLR4 polymorphisms in Southern Chinese Psoriasis Vulgaris patients. J Dermatol Sci. 2016;83(2):145-7.

81. Xue X, Qiu Y, Jiang D, Jin T, Yan M, Zhu X, et al. The association analysis of TLR2 and TLR4 gene with tuberculosis in the Tibetan Chinese population. Oncotarget. 2017;8(68):113082-9.

82. Bjornvold M, Munthe-Kaas MC, Egeland T, Joner G, Dahl-Jorgensen K, Njolstad PR, et al. A TLR2 polymorphism is associated with type 1 diabetes and allergic asthma. Genes Immun. 2009;10(2):181-7.

83. Takahashi M, Chen Z, Watanabe K, Kobayashi H, Nakajima T, Kimura A, et al. Toll-like receptor 2 gene polymorphisms associated with aggressive periodontitis in Japanese. Open Dent J. 2011;5:190-4.

84. Purdue MP, Lan Q, Wang SS, Kricker A, Menashe I, Zheng TZ, et al. A pooled investigation of Toll-like receptor gene variants and risk of non-Hodgkin lymphoma. Carcinogenesis. 2009;30(2):275-81.

85. Ovsyannikova IG, Haralambieva IH, Vierkant RA, Pankratz VS, Jacobson RM, Poland GA. The role of polymorphisms in Toll-like receptors and their associated intracellular signaling genes in measles vaccine immunity. Human genetics. 2011;130(4):547-61.

86. Wu L, Hu Y, Li D, Jiang W, Xu B. Screening toll-like receptor markers to predict latent tuberculosis infection and subsequent tuberculosis disease in a Chinese population. BMC Med Genet. 2015;16:19.

87. de Barros Gallo C, Marichalar-Mendia X, Setien-Olarra A, Acha-Sagredo A, Bediaga NG, Gainza-Cirauqui ML, et al. Toll-like receptor 2 rs4696480 polymorphism and risk of oral cancer and oral potentially malignant disorder. Arch Oral Biol. 2017;82:109-14.

88. Sabah-Ozcan S, Gurel G. The polymorphism rs4696480 in the TLR2 gene is associated with psoriasis patients in the Turkish population. Immunol Lett. 2019;211:28-32.

89. Rosentul DC, Delsing CE, Jaeger M, Plantinga TS, Oosting M, Costantini I, et al. Gene polymorphisms in pattern recognition receptors and susceptibility to idiopathic recurrent vulvovaginal candidiasis. Front Microbiol. 2014;5:483.

90. Schroder NW, Diterich I, Zinke A, Eckert J, Draing C, von Baehr V, et al. Heterozygous Arg753Gln polymorphism of human TLR-2 impairs immune activation by Borrelia burgdorferi and protects from late stage Lyme disease. J Immunol. 2005;175(4):2534-40.

91. Tsui FW, Xi N, Rohekar S, Riarh R, Bilotta R, Tsui HW, et al. Toll-like receptor 2 variants are associated with acute reactive arthritis. Arthritis Rheum. 2008;58(11):3436-8.

92. Fischer M, Spies-Weisshart B, Schrenk K, Gruhn B, Wittig S, Glaser A, et al. Polymorphisms of Dectin-1 and TLR2 Predispose to Invasive Fungal Disease in Patients with Acute Myeloid Leukemia. PloS one. 2016;11(3):e0150632.

93. Zukowski M, Taryma-Lesniak O, Kaczmarczyk M, Kotfis K, Szydlowski L, Ciechanowicz A, et al. Relationship between toll-like receptor 2 R753Q and T16934A polymorphisms and Staphylococcus aureus nasal carriage. Anaesthesiol Intensive Ther. 2017;49(2):110-5.

94. Lee J, Choi J, Chung S, Park J, Kim JE, Sung H, et al. Genetic Predisposition of Polymorphisms in HMGB1-Related Genes to Breast Cancer Prognosis in Korean Women. J Breast Cancer. 2017;20(1):27-34.

95. Kaiser R, Tang LF, Taylor KE, Sterba K, Nititham J, Brown EE, et al. A polymorphism in TLR2 is associated with arterial thrombosis in a multiethnic population of patients with systemic lupus erythematosus. Arthritis Rheumatol. 2014;66(7):1882-7.

96. Jiang ZC, Tang XM, Zhao YR, Zheng L. A functional variant at miR-34a binding site in toll-like receptor 4 gene alters susceptibility to hepatocellular carcinoma in a Chinese Han population. Tumour biology : the journal of the International Society for Oncodevelopmental Biology and Medicine. 2014;35(12):12345-52.

97. Hwang YH, Ro H, Choi I, Kim H, Oh KH, Hwang JI, et al. Impact of polymorphisms of TLR4/CD14 and TLR3 on acute rejection in kidney transplantation. Transplantation. 2009;88(5):699-705.

98. Cheng I, Plummer SJ, Casey G, Witte JS. Toll-like receptor 4 genetic variation and advanced prostate cancer risk. Cancer epidemiology, biomarkers & prevention : a publication of the American Association for Cancer Research, cosponsored by the American Society of Preventive Oncology. 2007;16(2):352-5.

99. Castano-Rodriguez N, Kaakoush NO, Pardo AL, Goh KL, Fock KM, Mitchell HM. Genetic polymorphisms in the Toll-like receptor signalling pathway in Helicobacter pylori infection and related gastric cancer. Hum Immunol. 2014;75(8):808-15.

100. Fan YF, Wu YM, Liu H, Yu Y, Jiang YY, Xue YZ, et al. TLR4 polymorphisms associated with developing gastric pre-cancer lesions in a Chinese Han population. Hum Immunol. 2014;75(2):176-81.

101. Li P, He CY, Xu Q, Sun LP, Ha MW, Yuan Y. Effect of the -2081G/A polymorphism of the TLR4 gene and its interaction with Helicobacter pylori infection on the risk of gastric cancer in Chinese individuals. Genetic testing and molecular biomarkers. 2014;18(9):610-5.

102. Ito M, Hanaoka M, Droma Y, Kobayashi N, Yasuo M, Kitaguchi Y, et al. The association of Toll-like receptor 4 gene polymorphisms with the development of emphysema in Japanese subjects: a case control study. BMC research notes. 2012;5:36.

103. Zhou L, Wei B, Xing C, Xie H, Yu X, Wu L, et al. Polymorphism in 3'-untranslated region of toll-like receptor 4 gene is associated with protection from hepatitis B virus recurrence after liver transplantation. Transpl Infect Dis. 2011;13(3):250-8.

104. Miedema KG, te Poele EM, Tissing WJ, Postma DS, Koppelman GH, de Pagter AP, et al. Association of polymorphisms in the TLR4 gene with the risk of developing neutropenia in children with leukemia. Leukemia. 2011;25(6):995-1000.

105. Kim HJ, Bae JS, Chang IH, Kim KD, Lee J, Shin HD, et al. Sequence variants of Toll-like receptor 4 (TLR4) and the risk of prostate cancer in Korean men. World J Urol. 2012;30(2):225-32.

106. Mansur A, von Gruben L, Popov AF, Steinau M, Bergmann I, Ross D, et al. The regulatory toll-like receptor 4 genetic polymorphism rs11536889 is associated with renal, coagulation and hepatic organ failure in sepsis patients. J Transl Med. 2014;12:177.

107. Wang H, Wei Y, Zeng Y, Qin Y, Xiong B, Qin G, et al. The association of polymorphisms of TLR4 and CD14 genes with susceptibility to sepsis in a Chinese population. BMC Med Genet. 2014;15:123.

108. Liu L, Zhang Q, Xiong XY, Gong QW, Liao MF, Yang QW. TLR4 gene polymorphisms rs11536889 is associated with intracranial aneurysm susceptibility. Journal of clinical neuroscience : official journal of the Neurosurgical Society of Australasia. 2018;53:165-70.

109. Shibuya E, Meguro A, Ota M, Kashiwagi K, Mabuchi F, Iijima H, et al. Association of Toll-like receptor 4 gene polymorphisms with normal tension glaucoma. Investigative ophthalmology & visual science. 2008;49(10):4453-7.

110. Ryckman KK, Williams SM, Krohn MA, Simhan HN. Genetic association of Toll-like receptor 4 with cervical cytokine concentrations during pregnancy. Genes Immun. 2009;10(7):636-40.

111. Sode J, Bank S, Vogel U, Andersen PS, Sorensen SB, Bojesen AB, et al. Genetically determined high activities of the TNF-alpha, IL23/IL17, and NFkB pathways were associated with increased risk of ankylosing spondylitis. BMC Med Genet. 2018;19(1):165.

112. Chen YC, Yip PK, Huang YL, Sun Y, Wen LL, Chu YM, et al. Sequence variants of toll like receptor 4 and late-onset Alzheimer's disease. PloS one. 2012;7(12):e50771.

113. Enquobahrie DA, Smith NL, Bis JC, Carty CL, Rice KM, Lumley T, et al. Cholesterol ester transfer protein, interleukin-8, peroxisome proliferator activator receptor alpha, and Toll-like receptor 4 genetic variations and risk of incident nonfatal myocardial infarction and ischemic stroke. Am J Cardiol. 2008;101(12):1683-8.

114. Song J, Kim DY, Kim CS, Kim HJ, Lee DH, Lee HM, et al. The association between Toll-like receptor 4 (TLR4) polymorphisms and the risk of prostate cancer in Korean men. Cancer genetics and cytogenetics. 2009;190(2):88-92.

115. Taylor BD, Darville T, Ferrell RE, Kammerer CM, Ness RB, Haggerty CL. Variants in toll-like receptor 1 and 4 genes are associated with Chlamydia trachomatis among women with pelvic inflammatory disease. J Infect Dis. 2012;205(4):603-9.

116. Huang L, Yuan K, Liu J, Ren X, Dong X, Tian W, et al. Polymorphisms of the TLR4 gene and risk of gastric cancer. Gene. 2014;537(1):46-50.

117. Zaki HY, Leung KH, Yiu WC, Gasmelseed N, Elwali NE, Yip SP. Common polymorphisms in TLR4 gene associated with susceptibility to pulmonary tuberculosis in the Sudanese. Int J Tuberc Lung Dis. 2012;16(7):934-40.

118. Song Y, Liu H, Long L, Zhang N, Liu Y. TLR4 rs1927911, but not TLR2 rs5743708, is associated with atherosclerotic cerebral infarction in the Southern Han population: a case-control study. Medicine (Baltimore). 2015;94(2):e381.

119. Xu Y, Jiang Z, Huang J, Meng Q, Coh P, Tao L. The association between toll-like receptor 4 polymorphisms and diabetic retinopathy in Chinese patients with type 2 diabetes. Br J Ophthalmol. 2015;99(9):1301-5.

120. Zhao J, Han X, Xue L, Zhu K, Liu H, Xie A. Association of TLR4 gene polymorphisms with sporadic Parkinson's disease in a Han Chinese population. Neurol Sci. 2015;36(9):1659-65.

121. Li Z, Song A, Yu H. Interaction between toll-like receptor 4 (TLR4) gene and alcohol drinking on Parkinson's disease risk in Chinese Han population. Journal of clinical neuroscience : official journal of the Neurosurgical Society of Australasia. 2019;62:128-32.

122. Qing YF, Zhou JG, Zhang QB, Wang DS, Li M, Yang QB, et al. Association of TLR4 Gene rs2149356 polymorphism with primary gouty arthritis in a case-control study. PloS one. 2013;8(5):e64845.

123. Takano Y, Shi D, Shimizu A, Funayama T, Mashima Y, Yasuda N, et al. Association of Toll-like receptor 4 gene polymorphisms in Japanese subjects with primary open-angle, normal-tension, and exfoliation glaucoma. Am J Ophthalmol. 2012;154(5):825-32 e1.

124. Rasheed H, McKinney C, Stamp LK, Dalbeth N, Topless RK, Day R, et al. The Toll-Like Receptor 4 (TLR4) Variant rs2149356 and Risk of Gout in European and Polynesian Sample Sets. PloS one. 2016;11(1):e0147939.

125. Liu H, Qi S, He W, Chang C, Chen Y, Yu J. Association of single-nucleotide polymorphisms in TLR4 gene and gene-environment interaction with primary open angle glaucoma in a Chinese northern population. J Gene Med. 2020;22(1):e3139.

126. Yang H, Wei C, Li Q, Shou T, Yang Y, Xiao C, et al. Association of TLR4 gene non-missense single nucleotide polymorphisms with rheumatoid arthritis in Chinese Han population. Rheumatol Int. 2013;33(5):1283-8.

127. Rudofsky G, Jr., Reismann P, Witte S, Humpert PM, Isermann B, Chavakis T, et al. Asp299Gly and Thr399Ile genotypes of the TLR4 gene are associated with a reduced prevalence of diabetic neuropathy in patients with type 2 diabetes. Diabetes care. 2004;27(1):179-83.

128. Ameziane N, Beillat T, Verpillat P, Chollet-Martin S, Aumont MC, Seknadji P, et al. Association of the Toll-like receptor 4 gene Asp299Gly polymorphism with acute coronary events. Arterioscler Thromb Vasc Biol. 2003;23(12):e61-4.

129. Kiechl S, Lorenz E, Reindl M, Wiedermann CJ, Oberhollenzer F, Bonora E, et al. Toll-like receptor 4 polymorphisms and atherogenesis. The New England journal of medicine. 2002;347(3):185-92.

130. Agnese DM, Calvano JE, Hahm SJ, Coyle SM, Corbett SA, Calvano SE, et al. Human toll-like receptor 4 mutations but not CD14 polymorphisms are associated with an increased risk of gram-negative infections. J Infect Dis. 2002;186(10):1522-5.

131. Tal G, Mandelberg A, Dalal I, Cesar K, Somekh E, Tal A, et al. Association between common Toll-like receptor 4 mutations and severe respiratory syncytial virus disease. J Infect Dis. 2004;189(11):2057-63.

132. Palmer SM, Burch LH, Davis RD, Herczyk WF, Howell DN, Reinsmoen NL, et al. The role of innate immunity in acute allograft rejection after lung transplantation. Am J Respir Crit Care Med. 2003;168(6):628-32.

133. Palomino-Morales R, Torres O, Vazquez-Rodriguez TR, Morado IC, Castaneda S, Callejas-Rubio JL, et al. Association between toll-like receptor 4 gene polymorphism and biopsy-proven giant cell arteritis. J Rheumatol. 2009;36(7):1501-6.

134. da Silva Santos S, Clark TG, Campino S, Suarez-Mutis MC, Rockett KA, Kwiatkowski DP, et al. Investigation of host candidate malaria-associated risk/protective SNPs in a Brazilian Amazonian population. PloS one. 2012;7(5):e36692.

135. Dhiman N, Ovsyannikova IG, Vierkant RA, Ryan JE, Pankratz VS, Jacobson RM, et al. Associations between SNPs in toll-like receptors and related intracellular signaling molecules and immune responses to measles vaccine: preliminary results. Vaccine. 2008;26(14):1731-6.

136. van Rijn BB, Franx A, Steegers EA, de Groot CJ, Bertina RM, Pasterkamp G, et al. Maternal TLR4 and NOD2 gene variants, pro-inflammatory phenotype and susceptibility to early-onset preeclampsia and HELLP syndrome. PloS one. 2008;3(4):e1865.

137. Hawn TR, Scholes D, Li SS, Wang H, Yang Y, Roberts PL, et al. Toll-like receptor polymorphisms and susceptibility to urinary tract infections in adult women. PloS one. 2009;4(6):e5990.

138. Cussigh A, Fabris C, Fattovich G, Falleti E, Cmet S, Bitetto D, et al. Toll like receptor 4 D299G associates with disease progression in Caucasian patients with chronic HBV infection: relationship with gender. J Clin Immunol. 2013;33(2):313-6.

139. de Oliveira JG, Rossi AF, Nizato DM, Miyasaki K, Silva AE. Profiles of gene polymorphisms in cytokines and Toll-like receptors with higher risk for gastric cancer. Digestive diseases and sciences. 2013;58(4):978-88.

140. Sampath V, Mulrooney NP, Garland JS, He J, Patel AL, Cohen JD, et al. Toll-like receptor genetic variants are associated with Gram-negative infections in VLBW infants. J Perinatol. 2013;33(10):772-7.

141. Wu JF, Chen CH, Ni YH, Lin YT, Chen HL, Hsu HY, et al. Toll-like receptor and hepatitis B virus clearance in chronic infected patients: a long-term prospective cohort study in Taiwan. J Infect Dis. 2012;206(5):662-8.

142. Wagner J, Skinner NA, Catto-Smith AG, Cameron DJ, Michalski WP, Visvanathan K, et al. TLR4, IL10RA, and NOD2 mutation in paediatric Crohn's disease patients: an association with Mycobacterium avium subspecies paratuberculosis and TLR4 and IL10RA expression. Med Microbiol Immunol. 2013;202(4):267-76.

143. Al-Qahtani AA, Al-Anazi MR, Al-Zoghaibi F, Abdo AA, Sanai FM, Khan MQ, et al. The association of toll-like receptor 4 polymorphism with hepatitis C virus infection in Saudi Arabian patients. BioMed research international. 2014;2014:357062.

144. Carvalho A, Pasqualotto AC, Pitzurra L, Romani L, Denning DW, Rodrigues F. Polymorphisms in toll-like receptor genes and susceptibility to pulmonary aspergillosis. J Infect Dis. 2008;197(4):618-21.

145. Gu X, Shen Y, Fu L, Zuo HY, Yasen H, He P, et al. Polymorphic variation of inflammation-related genes and risk of non-Hodgkin lymphoma for Uygur and Han Chinese in Xinjiang. Asian Pac J Cancer Prev. 2014;15(21):9177-83.

146. Hafren L, Einarsdottir E, Kentala E, Hammaren-Malmi S, Bhutta MF, MacArthur CJ, et al. Predisposition to Childhood Otitis Media and Genetic Polymorphisms within the Toll-Like Receptor 4 (TLR4) Locus. PloS one. 2015;10(7):e0132551.

147. Tsilidis KK, Helzlsouer KJ, Smith MW, Grinberg V, Hoffman-Bolton J, Clipp SL, et al. Association of common polymorphisms in IL10, and in other genes related to inflammatory response and obesity with colorectal cancer. Cancer Causes Control. 2009;20(9):1739-51.

148. Chen Z, Nakajima T, Inoue Y, Kudo T, Jibiki M, Iwai T, et al. A single nucleotide polymorphism in the 3'-untranslated region of MyD88 gene is associated with Buerger disease but not with Takayasu arteritis in Japanese. Journal of human genetics. 2011;56(7):545-7.

149. Potter C, Cordell HJ, Barton A, Daly AK, Hyrich KL, Mann DA, et al. Association between anti-tumour necrosis factor treatment response and genetic variants within the TLR and NF{kappa}B signalling pathways. Annals of the rheumatic diseases. 2010;69(7):1315-20.

150. Capparelli R, De Chiara F, Di Matteo A, Medaglia C, Iannelli D. The MyD88 rs6853 and TIRAP rs8177374 polymorphic sites are associated with resistance to human pulmonary tuberculosis. Genes Immun. 2013;14(8):504-11.

151. Sigurdson AJ, Brenner AV, Roach JA, Goudeva L, Muller JA, Nerlich K, et al. Selected single-nucleotide polymorphisms in FOXE1, SERPINA5, FTO, EVPL, TICAM1 and SCARB1 are associated with papillary and follicular thyroid cancer risk: replication study in a German population. Carcinogenesis. 2016;37(7):677-84.

152. Yang Y, Yang S, Chen Z, Liu L. Correlation between TICAM1 gene polymorphisms and community-acquired pneumonia in children. Journal of biochemical and molecular toxicology. 2020:e22503.

153. Kim SK, Jang HM, Kim DY. The promoter polymorphism of NFKB1 gene contributes to susceptibility of ischemic stroke in Korean population. J Exerc Rehabil. 2018;14(6):1096-100.

154. Chang ET, Birmann BM, Kasperzyk JL, Conti DV, Kraft P, Ambinder RF, et al. Polymorphic variation in NFKB1 and other aspirin-related genes and risk of Hodgkin lymphoma. Cancer epidemiology, biomarkers & prevention : a publication of the American Association for Cancer Research, cosponsored by the American Society of Preventive Oncology. 2009;18(3):976-86.

155. Gao J, Xu HL, Gao S, Zhang W, Tan YT, Rothman N, et al. Genetic polymorphism of NFKB1 and NFKBIA genes and liver cancer risk: a nested case-control study in Shanghai, China. BMJ Open. 2014;4(2):e004427.

156. Liou YJ, Wang HH, Lee MT, Wang SC, Chiang HL, Chen CC, et al. Genome-wide association study of treatment refractory schizophrenia in Han Chinese. PloS one. 2012;7(3):e33598.

157. Yang YN, Zhang JY, Ma YT, Xie X, Li XM, Liu F, et al. -94 ATTG insertion/deletion polymorphism of the NFKB1 gene is associated with coronary artery disease in Han and Uygur women in China. Genetic testing and molecular biomarkers. 2014;18(6):430-8.

158. Zhou B, Qie M, Wang Y, Yan L, Zhang Z, Liang A, et al. Relationship between NFKB1 -94 insertion/deletion ATTG polymorphism and susceptibility of cervical squamous cell carcinoma risk. Ann Oncol. 2010;21(3):506-11.

159. Andersen V, Christensen J, Overvad K, Tjonneland A, Vogel U. Polymorphisms in NFkB, PXR, LXR and risk of colorectal cancer in a prospective study of Danes. BMC cancer. 2010;10:484.

160. Gao M, Wang CH, Sima X, Han XM. NFKB1 -94 insertion/deletion ATTG polymorphism contributes to risk of systemic lupus erythematosus. DNA Cell Biol. 2012;31(4):611-5.

161. Zhou B, Rao L, Peng Y, Wang Y, Li Y, Gao L, et al. Functional polymorphism of the NFKB1 gene promoter is related to the risk of dilated cardiomyopathy. BMC Med Genet. 2009;10:47.

162. Mohd Suzairi MS, Tan SC, Ahmad Aizat AA, Mohd Aminudin M, Siti Nurfatimah MS, Andee ZD, et al. The functional -94 insertion/deletion ATTG polymorphism in the promoter region of NFKB1 gene increases the risk of sporadic colorectal cancer. Cancer Epidemiol. 2013;37(5):634-8.

163. Wang X, Peng H, Liang Y, Sun R, Wei T, Li Z, et al. A functional insertion/deletion polymorphism in the promoter region of the NFKB1 gene increases the risk of papillary thyroid carcinoma. Genetic testing and molecular biomarkers. 2015;19(3):167-71.

164. Oner T, Yenmis G, Tombulturk K, Cam C, Kucuk OS, Yakicier MC, et al. Association of Pre-miRNA-499 rs3746444 and Pre-miRNA-146a rs2910164 Polymorphisms and Susceptibility to Behcet's Disease. Genetic testing and molecular biomarkers. 2015;19(8):424-30.

165. Lai HM, Li XM, Yang YN, Ma YT, Xu R, Pan S, et al. Genetic Variation in NFKB1 and NFKBIA and Susceptibility to Coronary Artery Disease in a Chinese Uygur Population. PloS one. 2015;10(6):e0129144.

166. Lai H, Chen Q, Li X, Ma Y, Xu R, Zhai H, et al. Association between genetic polymorphism in NFKB1 and NFKBIA and coronary artery disease in a Chinese Han population. Int J Clin Exp Med. 2015;8(11):21487-96.

167. Guo XL, Liu XC, Su GB, Zhou CY, Cui QT. Association of NF-kappaB1 gene polymorphisms with coronary artery disease in a Han Chinese population. Genet Mol Res. 2016;15(3).

168. Fan HZ, Huang P, Shao JG, Tian T, Li J, Zang F, et al. Genetic variation on the NFKB1 genes associates with the outcomes of HCV infection among Chinese Han population. Infect Genet Evol. 2018;65:210-5.

169. Jin SY, Luo JY, Li XM, Liu F, Ma YT, Gao XM, et al. NFKB1 gene rs28362491 polymorphism is associated with the susceptibility of acute coronary syndrome. Biosci Rep. 2019;39(4).

170. Sapcharoen K, Sanguansermsri P, Yasothornsrikul S, Muisuk K, Srikummool M. Gene Combination of CD44 rs187116, CD133 rs2240688, NF-kappaB1 rs28362491 and GSTM1 Deletion as a Potential Biomarker in Risk Prediction of Breast Cancer in Lower Northern Thailand. Asian Pac J Cancer Prev. 2019;20(8):2493-502.

171. Vu D, Tellez-Corrales E, Sakharkar P, Kissen MS, Shah T, Hutchinson I, et al. Impact of NF-kappaB gene polymorphism on allograft outcome in Hispanic renal transplant recipients. Transpl Immunol. 2013;28(1):18-23.

172. Cerhan JR, Liu-Mares W, Fredericksen ZS, Novak AJ, Cunningham JM, Kay NE, et al. Genetic variation in tumor necrosis factor and the nuclear factor-kappaB canonical pathway and risk of non-Hodgkin's lymphoma. Cancer epidemiology, biomarkers & prevention : a publication of the American Association for Cancer Research, cosponsored by the American Society of Preventive Oncology. 2008;17(11):3161-9.

173. Shiels MS, Engels EA, Shi J, Landi MT, Albanes D, Chatterjee N, et al. Genetic variation in innate immunity and inflammation pathways associated with lung cancer risk. Cancer. 2012;118(22):5630-6.

174. Manuel Sanchez-Maldonado J, Martinez-Bueno M, Canhao H, Ter Horst R, Munoz-Pena S, Moniz-Diez A, et al. NFKB2 polymorphisms associate with the risk of developing rheumatoid arthritis and response to TNF inhibitors: Results from the REPAIR consortium. Sci Rep. 2020;10(1):4316.

175. Pare G, Ridker PM, Rose L, Barbalic M, Dupuis J, Dehghan A, et al. Genome-wide association analysis of soluble ICAM-1 concentration reveals novel associations at the NFKBIK, PNPLA3, RELA, and SH2B3 loci. PLoS genetics. 2011;7(4):e1001374.

176. Bowes J, Ho P, Flynn E, Ali F, Marzo-Ortega H, Coates LC, et al. Comprehensive assessment of rheumatoid arthritis susceptibility loci in a large psoriatic arthritis cohort. Annals of the rheumatic diseases. 2012;71(8):1350-4.

177. Gregersen PK, Amos CI, Lee AT, Lu Y, Remmers EF, Kastner DL, et al. REL, encoding a member of the NF-kappaB family of transcription factors, is a newly defined risk locus for rheumatoid arthritis. Nature genetics. 2009;41(7):820-3.

178. Eyre S, Hinks A, Flynn E, Martin P, Wilson AG, Maxwell JR, et al. Confirmation of association of the REL locus with rheumatoid arthritis susceptibility in the UK population. Annals of the rheumatic diseases. 2010;69(8):1572-3.

179. Ali FR, Barton A, Smith RL, Bowes J, Flynn E, Mangino M, et al. An investigation of rheumatoid arthritis loci in patients with early-onset psoriasis validates association of the REL gene. Br J Dermatol. 2013;168(4):864-6.

180. Trynka G, Zhernakova A, Romanos J, Franke L, Hunt KA, Turner G, et al. Coeliac disease-associated risk variants in TNFAIP3 and REL implicate altered NF-kappaB signalling. Gut. 2009;58(8):1078-83.

181. Li F, Xu J, Zheng J, Sokolove J, Zhu K, Zhang Y, et al. Association between interleukin-6 gene polymorphisms and rheumatoid arthritis in Chinese Han population: a case-control study and a meta-analysis. Sci Rep. 2014;4:5714.

182. Cai J, Cui K, Niu F, Jin T, Huang S, Zhang Y, et al. Genetics of IL6 polymorphisms: Case-control study of the risk of endometrial cancer. Mol Genet Genomic Med. 2019;7(4):e00600.

183. Li H, Dai H, Li H, Li B, Shao Y. Polymorphisms of the Highly Expressed IL-6 Gene in the Papillary Thyroid Cancer Susceptibility Among Chinese. Current molecular medicine. 2019;19(6):443-51.

184. Illig T, Bongardt F, Schopfer A, Muller-Scholze S, Rathmann W, Koenig W, et al. Significant association of the interleukin-6 gene polymorphisms C-174G and A-598G with type 2 diabetes. The Journal of clinical endocrinology and metabolism. 2004;89(10):5053-8.

185. Bamoulid J, Courivaud C, Deschamps M, Mercier P, Ferrand C, Penfornis A, et al. IL-6 promoter polymorphism -174 is associated with new-onset diabetes after transplantation. J Am Soc Nephrol. 2006;17(8):2333-40.

186. Lu KC, Jaramillo A, Lecha RL, Schuessler RB, Aloush A, Trulock EP, et al. Interleukin-6 and interferon-gamma gene polymorphisms in the development of bronchiolitis obliterans syndrome after lung transplantation. Transplantation. 2002;74(9):1297-302.

187. Foster CB, Lehrnbecher T, Samuels S, Stein S, Mol F, Metcalf JA, et al. An IL6 promoter polymorphism is associated with a lifetime risk of development of Kaposi sarcoma in men infected with human immunodeficiency virus. Blood. 2000;96(7):2562-7.

188. Antonicelli R, Olivieri F, Bonafe M, Cavallone L, Spazzafumo L, Marchegiani F, et al. The interleukin-6 -174 G>C promoter polymorphism is associated with a higher risk of death after an acute coronary syndrome in male elderly patients. Int J Cardiol. 2005;103(3):266-71.

189. Jeng JR, Wang JH, Liu WS, Chen SP, Chen MY, Wu MH, et al. Association of interleukin-6 gene G-174C polymorphism and plasma plasminogen activator inhibitor-1 level in Chinese patients with and without hypertension. Am J Hypertens. 2005;18(4 Pt 1):517-22.

190. Muller-Steinhardt M, Hartel C, Muller B, Kirchner H, Fricke L. The interleukin-6 -174promoter polymorphism is associated with long-term kidney allograft survival. Kidney Int. 2002;62(5):1824-7.

191. Grocott HP, White WD, Morris RW, Podgoreanu MV, Mathew JP, Nielsen DM, et al. Genetic polymorphisms and the risk of stroke after cardiac surgery. Stroke; a journal of cerebral circulation. 2005;36(9):1854-8.

192. Licastro F, Grimaldi LM, Bonafe M, Martina C, Olivieri F, Cavallone L, et al. Interleukin-6 gene alleles affect the risk of Alzheimer's disease and levels of the cytokine in blood and brain. Neurobiol Aging. 2003;24(7):921-6.

193. Hurme M, Lehtimaki T, Jylha M, Karhunen PJ, Hervonen A. Interleukin-6 -174G/C polymorphism and longevity: a follow-up study. Mech Ageing Dev. 2005;126(3):417-8.

194. Cole JW, Brown DW, Giles WH, Stine OC, O'Connell JR, Mitchell BD, et al. Ischemic stroke risk, smoking, and the genetics of inflammation in a biracial population: the stroke prevention in young women study. Thromb J. 2008;6:11.

195. O'Mahony DS, Glavan BJ, Holden TD, Fong C, Black RA, Rona G, et al. Inflammation and immune-related candidate gene associations with acute lung injury susceptibility and severity: a validation study. PloS one. 2012;7(12):e51104.

196. Shi TY, Zhu ML, He J, Wang MY, Li QX, Zhou XY, et al. Polymorphisms of the Interleukin 6 gene contribute to cervical cancer susceptibility in Eastern Chinese women. Human genetics. 2013;132(3):301-12.

197. He S, Yang S, Zhao Q, Wang L, Liu H, Sheng Y, et al. Association of IL4, IL6, and IL10 polymorphisms with pulmonary tuberculosis in a Tibetan Chinese population. Oncotarget. 2018;9(23):16418-26.

198. Tabassum R, Mahendran Y, Dwivedi OP, Chauhan G, Ghosh S, Marwaha RK, et al. Common variants of IL6, LEPR, and PBEF1 are associated with obesity in Indian children. Diabetes. 2012;61(3):626-31.

199. Hussain S, Iqbal T, Sadiq I, Feroz S, Shafique Satti H. Polymorphism in the IL-8 Gene Promoter and the Risk of Acne Vulgaris in a Pakistani Population. Iran J Allergy Asthma Immunol. 2015;14(4):443-9.

200. Velasquez IM, Frumento P, Johansson K, Berglund A, de Faire U, Leander K, et al. Association of interleukin 8 with myocardial infarction: results from the Stockholm Heart Epidemiology Program. Int J Cardiol. 2014;172(1):173-8.

201. Rafrafi A, Chahed B, Kaabachi S, Kaabachi W, Maalmi H, Hamzaoui K, et al. Association of IL-8 gene polymorphisms with non small cell lung cancer in Tunisia: A case control study. Hum Immunol. 2013;74(10):1368-74.

202. Ahn MH, Park BL, Lee SH, Park SW, Park JS, Kim DJ, et al. A promoter SNP rs4073T>A in the common allele of the interleukin 8 gene is associated with the development of idiopathic pulmonary fibrosis via the IL-8 protein enhancing mode. Respir Res. 2011;12:73.

203. Suh JS, Hahn WH, Cho BS. Polymorphisms of CXCL8 and its receptor CXCR2 contribute to the development and progression of childhood IgA nephropathy. J Interferon Cytokine Res. 2011;31(3):309-15.

204. Dan H, Liu W, Zhou Y, Wang J, Chen Q, Zeng X. Association of interleukin-8 gene polymorphisms and haplotypes with oral lichen planus in a Chinese population. Inflammation. 2010;33(2):76-81.

205. Cerhan JR, Wang S, Maurer MJ, Ansell SM, Geyer SM, Cozen W, et al. Prognostic significance of host immune gene polymorphisms in follicular lymphoma survival. Blood. 2007;109(12):5439-46.

206. Charrad R, Kaabachi W, Rafrafi A, Berraies A, Hamzaoui K, Hamzaoui A. IL-8 Gene Variants and Expression in Childhood Asthma. Lung. 2017;195(6):749-57.

207. Gonzalez-Hormazabal P, Romero S, Musleh M, Bustamante M, Stambuk J, Pisano R, et al. IL-8-251T>A (rs4073) Polymorphism Is Associated with Prognosis in Gastric Cancer Patients. Anticancer Res. 2018;38(10):5703-8.

208. Gu LQ, Jia HY, Zhao YJ, Liu N, Wang S, Cui B, et al. Association studies of interleukin-8 gene in Graves' disease and Graves' ophthalmopathy. Endocrine. 2009;36(3):452-6.

209. Scarel-Caminaga RM, Kim YJ, Viana AC, Curtis KM, Corbi SC, Sogumo PM, et al. Haplotypes in the interleukin 8 gene and their association with chronic periodontitis susceptibility. Biochem Genet. 2011;49(5-6):292-302.

210. Lupianez CB, Canet LM, Carvalho A, Alcazar-Fuoli L, Springer J, Lackner M, et al. Polymorphisms in Host Immunity-Modulating Genes and Risk of Invasive Aspergillosis: Results from the AspBIOmics Consortium. Infect Immun. 2015;84(3):643-57.
