# Supplementary material for: Germline Genetic Variants of Viral Entry and Innate Immunity May Influence Susceptibility to SARS-CoV-2 Infection: Toward a Polygenic Risk Score for Risk Stratification
Source: Front Immunol. 2021 Mar 8;12:653489. doi: 10.3389/fimmu.2021.653489 (PMC7982482; doi:10.3389/fimmu.2021.653489)
Supplement: Supplementary file 2 [file Table_2.DOCX]

Supplementary Material

# Supplementary Table 2

**Minor allele frequencies (MAFs) of SNPs from Table 1 and Supplementary Table 1 in different populations highlight population-specific MAFs** Light blue background in column 1 and 2 highlight genes and SNPs from Table 1 (SNPs of viral entry), while additional genes and SNPs are from Supplementary Table 1 (SNPs of innate immunity). All data presented here were downloaded from the gnomAD database (gnomad.broadinstitute.org). MAFs in East Asian (EAS), Non-Finnish European (NFE), Latino/Admixed American (AMR) and African/African American (AFR) superpopulations are presented, followed by global MAF data (XX: female, XY: male, average). Variance was calculated from the presented 4 superpopulation data. Red color coding correspond to MAFs.

| Gene ID | SNP ID | **EAS** | **NFE** | **AMR** | **AFR** | **XX** | **XY** | **Average** | **Variance** |
| --- | --- | --- | --- | --- | --- | --- | --- | --- | --- |
| NFKB2 | rs11574851 | 0.065 | 0.051 | 0.034 | 0.010 | 0.038 | 0.038 | 0.038 | 0.0004 |
| IRF7 | rs1131665 | 0.026 | 0.270 | 0.307 | 0.519 | 0.335 | 0.314 | 0.325 | 0.0306 |
| IRF7 | rs1061501 | 0.737 | 0.867 | 0.764 | 0.843 | 0.833 | 0.838 | 0.836 | 0.0029 |
| IRF7 | rs3758650 | 0.092 | 0.057 | 0.027 | 0.017 | 0.045 | 0.051 | 0.048 | 0.0009 |
| RELA | rs1049728 | 0.001 | 0.065 | 0.031 | 0.010 | 0.045 | 0.053 | 0.049 | 0.0006 |
| TPCN2 | rs35264875 | 0.004 | 0.185 | 0.060 | 0.032 | 0.147 | 0.167 | 0.157 | 0.0048 |
| TPCN2 | rs1551305 | 0.289 | 0.488 | 0.348 | 0.415 | 0.455 | 0.448 | 0.451 | 0.0056 |
| TPCN2 | rs3829241 | 0.219 | 0.389 | 0.254 | 0.096 | 0.282 | 0.272 | 0.277 | 0.0109 |
| FURIN | rs17514846 | 0.166 | 0.463 | 0.268 | 0.813 | 0.522 | 0.530 | 0.526 | 0.0609 |
| FURIN | rs4702 | 0.527 | 0.568 | 0.574 | 0.882 | 0.640 | 0.648 | 0.644 | 0.0202 |
| TRIF/TICAM1 | rs8120 | 0.424 | 0.244 | 0.301 | 0.249 | 0.260 | 0.273 | 0.267 | 0.0053 |
| TRIF/TICAM1 | rs11466711 | 0.312 | 0.158 | 0.212 | 0.114 | 0.158 | 0.170 | 0.164 | 0.0055 |
| IRF3 | rs2304206 | 0.079 | 0.139 | 0.044 | 0.016 | 0.091 | 0.087 | 0.089 | 0.0021 |
| IRF3 | rs2304204 | 0.065 | 0.000 | 0.033 | 0.001 | 0.006 | 0.006 | 0.006 | 0.0007 |
| REL | rs842647 | 0.132 | 0.677 | 0.537 | 0.600 | 0.623 | 0.608 | 0.615 | 0.0443 |
| REL | rs13031237 | 0.020 | 0.367 | 0.233 | 0.090 | 0.257 | 0.245 | 0.251 | 0.0178 |
| REL | rs13017599 | 0.016 | 0.364 | 0.232 | 0.090 | 0.256 | 0.244 | 0.250 | 0.0179 |
| MDA5/IFIH1 | rs1990760 | 0.195 | 0.610 | 0.443 | 0.186 | 0.455 | 0.449 | 0.452 | 0.0317 |
| MDA5/IFIH1 | rs35667974 | 0.000 | 0.019 | 0.003 | 0.003 | 0.012 | 0.011 | 0.011 | 0.0001 |
| MDA5/IFIH1 | rs3747517 | 0.321 | 0.728 | 0.776 | 0.622 | 0.683 | 0.671 | 0.677 | 0.0313 |
| MDA5/IFIH1 | rs78456138 | 0.000 | 0.022 | 0.006 | 0.006 | 0.014 | 0.013 | 0.013 | 0.0001 |
| MDA5/IFIH1 | rs35744605 | 0.000 | 0.000 | 0.000 | 0.000 | 0.000 | 0.000 | 0.000 | 0.0000 |
| MDA5/IFIH1 | rs35337543 | 0.000 | 0.000 | 0.000 | 0.000 | 0.000 | 0.000 | 0.000 | 0.0000 |
| MDA5/IFIH1 | rs10930046 | 0.124 | 0.013 | 0.250 | 0.415 | 0.153 | 0.151 | 0.152 | 0.0224 |
| MDA5/IFIH1 | rs13023380 | 0.008 | 0.517 | 0.362 | 0.097 | 0.358 | 0.347 | 0.352 | 0.0414 |
| MAVS | rs11905552 | 0.000 | 0.001 | 0.007 | 0.130 | 0.016 | 0.010 | 0.013 | 0.0030 |
| MAVS | rs7269320 | 0.085 | 0.157 | 0.154 | 0.371 | 0.215 | 0.205 | 0.210 | 0.0116 |
| TMPRSS2 | rs2070788 | 0.652 | 0.533 | 0.496 | 0.697 | 0.583 | 0.590 | 0.586 | 0.0068 |
| TMPRSS2 | rs12329760 | 0.387 | 0.228 | 0.157 | 0.288 | 0.246 | 0.258 | 0.252 | 0.0071 |
| TMPRSS2 | rs383510 | 0.674 | 0.515 | 0.593 | 0.650 | 0.584 | 0.575 | 0.579 | 0.0037 |
| MYD88 | rs4988453 | 0.012 | 0.049 | 0.031 | 0.090 | 0.061 | 0.061 | 0.061 | 0.0008 |
| MYD88 | rs7744 | 0.362 | 0.162 | 0.188 | 0.043 | 0.149 | 0.139 | 0.144 | 0.0130 |
| MYD88 | rs6853 | 0.026 | 0.122 | 0.114 | 0.257 | 0.155 | 0.154 | 0.155 | 0.0068 |
| TLR9 | rs352140 | 0.000 | 0.000 | 0.000 | 0.000 | 0.000 | 0.000 | 0.000 | 0.0000 |
| TLR9 | rs352139 | 0.369 | 0.551 | 0.545 | 0.577 | 0.524 | 0.510 | 0.517 | 0.0068 |
| TLR9 | rs5743836 | 0.004 | 0.150 | 0.128 | 0.351 | 0.198 | 0.183 | 0.190 | 0.0154 |
| TLR9 | rs187084 | 0.355 | 0.414 | 0.423 | 0.297 | 0.385 | 0.370 | 0.377 | 0.0026 |
| CXCL8 | rs2227532 | 0.000 | 0.004 | 0.019 | 0.082 | 0.028 | 0.027 | 0.027 | 0.0011 |
| CXCL8 | rs4073 | 0.601 | 0.545 | 0.609 | 0.207 | 0.472 | 0.454 | 0.463 | 0.0274 |
| CXCL8 | rs2227307 | 0.400 | 0.451 | 0.358 | 0.499 | 0.450 | 0.455 | 0.452 | 0.0028 |
| CXCL8 | rs2227306 | 0.331 | 0.420 | 0.298 | 0.133 | 0.320 | 0.311 | 0.315 | 0.0108 |
| NFKB1 | rs11940017 | 0.052 | 0.047 | 0.062 | 0.322 | 0.127 | 0.122 | 0.125 | 0.0135 |
| NFKB1 | rs28362491 | 0.402 | 0.391 | 0.485 | 0.494 | 0.428 | 0.429 | 0.428 | 0.0022 |
| NFKB1 | rs3774937 | 0.356 | 0.334 | 0.452 | 0.076 | 0.268 | 0.268 | 0.268 | 0.0194 |
| NFKB1 | rs1585215 | 0.351 | 0.330 | 0.406 | 0.079 | 0.264 | 0.273 | 0.269 | 0.0159 |
| NFKB1 | rs230529 | 0.500 | 0.597 | 0.498 | 0.586 | 0.589 | 0.579 | 0.584 | 0.0022 |
| NFKB1 | rs230496 | 0.526 | 0.605 | 0.519 | 0.602 | 0.595 | 0.590 | 0.593 | 0.0016 |
| NFKB1 | rs4648022 | 0.000 | 0.000 | 0.000 | 0.000 | 0.000 | 0.000 | 0.000 | 0.0000 |
| NFKB1 | rs4699030 | 0.501 | 0.411 | 0.512 | 0.391 | 0.411 | 0.418 | 0.415 | 0.0028 |
| NFKB1 | rs4648127 | 0.049 | 0.057 | 0.030 | 0.011 | 0.037 | 0.041 | 0.039 | 0.0003 |
| TLR2 | rs893629 | 1.000 | 0.995 | 0.988 | 0.901 | 0.969 | 0.970 | 0.969 | 0.0016 |
| TLR2 | rs7696323 | 0.278 | 0.331 | 0.204 | 0.131 | 0.275 | 0.273 | 0.274 | 0.0057 |
| TLR2 | rs4696480 | 0.593 | 0.500 | 0.339 | 0.381 | 0.457 | 0.469 | 0.463 | 0.0100 |
| TLR2 | rs1898830 | 0.417 | 0.351 | 0.496 | 0.137 | 0.300 | 0.293 | 0.297 | 0.0179 |
| TLR2 | rs3804099 | 0.289 | 0.438 | 0.326 | 0.613 | 0.412 | 0.411 | 0.412 | 0.0158 |
| TLR2 | rs3804100 | 0.259 | 0.070 | 0.068 | 0.052 | 0.083 | 0.090 | 0.087 | 0.0072 |
| TLR2 | rs5743704 | 0.000 | 0.039 | 0.012 | 0.007 | 0.027 | 0.029 | 0.028 | 0.0002 |
| TLR2 | rs5743708 | 0.000 | 0.029 | 0.006 | 0.004 | 0.018 | 0.018 | 0.018 | 0.0001 |
| TLR2 | rs7656411 | 0.485 | 0.224 | 0.262 | 0.563 | 0.313 | 0.334 | 0.323 | 0.0207 |
| TLR3 | rs5743305 | 0.292 | 0.366 | 0.330 | 0.302 | 0.336 | 0.340 | 0.338 | 0.0008 |
| TLR3 | rs11721827 | 0.173 | 0.160 | 0.126 | 0.069 | 0.146 | 0.140 | 0.143 | 0.0016 |
| TLR3 | rs7657186 | 0.138 | 0.210 | 0.175 | 0.242 | 0.202 | 0.208 | 0.205 | 0.0015 |
| TLR3 | rs13126816 | 0.207 | 0.245 | 0.259 | 0.115 | 0.210 | 0.203 | 0.207 | 0.0032 |
| TLR3 | rs5743312 | 0.246 | 0.156 | 0.153 | 0.106 | 0.143 | 0.142 | 0.143 | 0.0026 |
| TLR3 | rs1879026 | 0.000 | 0.000 | 0.000 | 0.000 | 0.000 | 0.000 | 0.000 | 0.0000 |
| TLR3 | rs78726532 | 0.000 | 0.012 | 0.022 | 0.104 | 0.036 | 0.038 | 0.037 | 0.0017 |
| TLR3 | rs7668666 | 0.329 | 0.262 | 0.316 | 0.120 | 0.227 | 0.223 | 0.225 | 0.0069 |
| TLR3 | rs3775292 | 0.871 | 0.807 | 0.878 | 0.860 | 0.841 | 0.834 | 0.838 | 0.0008 |
| TLR3 | rs3775291 | 0.341 | 0.298 | 0.299 | 0.066 | 0.274 | 0.275 | 0.275 | 0.0117 |
| TLR3 | rs3775290 | 0.324 | 0.303 | 0.349 | 0.195 | 0.298 | 0.299 | 0.299 | 0.0034 |
| TLR3 | rs10025405 | 0.502 | 0.448 | 0.467 | 0.291 | 0.422 | 0.414 | 0.418 | 0.0065 |
| IL6 | rs1800795 | 1.000 | 0.557 | 0.809 | 0.927 | 0.666 | 0.696 | 0.681 | 0.0283 |
| IL6 | rs2069830 | 0.000 | 0.000 | 0.005 | 0.080 | 0.010 | 0.007 | 0.008 | 0.0012 |
| IL6 | rs2069832 | 1.000 | 0.558 | 0.810 | 0.926 | 0.667 | 0.696 | 0.682 | 0.0281 |
| IL6 | rs2069837 | 0.169 | 0.067 | 0.054 | 0.127 | 0.088 | 0.091 | 0.089 | 0.0021 |
| IL6 | rs1474347 | 0.992 | 0.558 | 0.802 | 0.840 | 0.643 | 0.671 | 0.657 | 0.0243 |
| IL6 | rs1524107 | 0.767 | 0.048 | 0.264 | 0.081 | 0.091 | 0.105 | 0.098 | 0.0826 |
| IL6 | rs2066992 | 0.765 | 0.048 | 0.267 | 0.081 | 0.091 | 0.105 | 0.098 | 0.0822 |
| IL6 | rs2069845 | 0.987 | 0.537 | 0.718 | 0.665 | 0.580 | 0.602 | 0.591 | 0.0270 |
| IFNA5 | rs3758236 | 0.465 | 0.179 | 0.282 | 0.265 | 0.223 | 0.221 | 0.222 | 0.0108 |
| IFNA8 | rs12553612 | 0.231 | 0.021 | 0.044 | 0.004 | 0.028 | 0.032 | 0.030 | 0.0083 |
| RIGI/DDX58 | rs3205166 | 0.495 | 0.364 | 0.342 | 0.231 | 0.347 | 0.342 | 0.345 | 0.0089 |
| RIGI/DDX58 | rs669260 | 0.000 | 0.000 | 0.000 | 0.000 | 0.000 | 0.000 | 0.000 | 0.0000 |
| RIGI/DDX58 | rs11795343 | 0.244 | 0.399 | 0.231 | 0.483 | 0.404 | 0.406 | 0.405 | 0.0113 |
| RIGI/DDX58 | rs10813831 | 0.104 | 0.261 | 0.140 | 0.234 | 0.213 | 0.216 | 0.214 | 0.0042 |
| RIGI/DDX58 | rs3739674 | 0.398 | 0.629 | 0.462 | 0.488 | 0.559 | 0.563 | 0.561 | 0.0071 |
| RIGI/DDX58 | rs56309110 | 0.000 | 0.000 | 0.000 | 0.000 | 0.000 | 0.000 | 0.000 | 0.0000 |
| CTSL | rs3118869 | 0.330 | 0.433 | 0.400 | 0.489 | 0.443 | 0.438 | 0.441 | 0.0033 |
| TLR4 | rs10983755 | 0.248 | 0.026 | 0.019 | 0.013 | 0.033 | 0.040 | 0.037 | 0.0099 |
| TLR4 | rs1927914 | 0.610 | 0.665 | 0.662 | 0.233 | 0.529 | 0.524 | 0.527 | 0.0325 |
| TLR4 | rs10759932 | 0.249 | 0.148 | 0.137 | 0.232 | 0.192 | 0.194 | 0.193 | 0.0024 |
| TLR4 | rs1927911 | 0.611 | 0.740 | 0.709 | 0.376 | 0.621 | 0.618 | 0.619 | 0.0204 |
| TLR4 | rs12377632 | 0.607 | 0.387 | 0.414 | 0.115 | 0.317 | 0.321 | 0.319 | 0.0307 |
| TLR4 | rs1927907 | 0.223 | 0.146 | 0.134 | 0.237 | 0.189 | 0.192 | 0.190 | 0.0021 |
| TLR4 | rs5030717 | 0.001 | 0.123 | 0.117 | 0.168 | 0.145 | 0.139 | 0.142 | 0.0038 |
| TLR4 | rs2149356 | 0.609 | 0.676 | 0.664 | 0.245 | 0.538 | 0.534 | 0.536 | 0.0314 |
| TLR4 | rs4986790 | 0.000 | 0.000 | 0.000 | 0.000 | 0.000 | 0.000 | 0.000 | 0.0000 |
| TLR4 | rs41426344 | 0.037 | 0.000 | 0.000 | 0.000 | 0.002 | 0.002 | 0.002 | 0.0002 |
| TLR4 | rs1057317 | N.A. | N.A. | N.A. | N.A. | N.A. | N.A. | N.A. | N.A. |
| TLR4 | rs11536889 | 0.248 | 0.140 | 0.103 | 0.031 | 0.107 | 0.110 | 0.108 | 0.0062 |
| TLR4 | rs7873784 | 0.122 | 0.150 | 0.087 | 0.212 | 0.157 | 0.159 | 0.158 | 0.0021 |
| TLR4 | rs11536898 | 0.119 | 0.126 | 0.073 | 0.205 | 0.140 | 0.142 | 0.141 | 0.0022 |
| TLR4 | rs1554973 | 0.173 | 0.243 | 0.176 | 0.713 | 0.360 | 0.374 | 0.367 | 0.0507 |
| TLR7 | rs5741880 | 0.039 | 0.091 | 0.092 | 0.424 | 0.180 | 0.174 | 0.177 | 0.0234 |
| TLR7 | rs179019 | 0.763 | 0.769 | 0.837 | 0.878 | 0.798 | 0.802 | 0.800 | 0.0023 |
| TLR7 | rs179010 | 0.681 | 0.698 | 0.851 | 0.842 | 0.733 | 0.734 | 0.734 | 0.0062 |
| TLR7 | rs179009 | 0.181 | 0.235 | 0.310 | 0.164 | 0.222 | 0.216 | 0.219 | 0.0032 |
| TLR7 | rs179008 | 0.000 | 0.218 | 0.189 | 0.129 | 0.181 | 0.177 | 0.179 | 0.0070 |
| TLR7 | rs3853839 | 0.792 | 0.164 | 0.483 | 0.185 | 0.210 | 0.224 | 0.217 | 0.0656 |
| TLR8 | rs3764879 | 0.847 | 0.234 | 0.515 | 0.278 | 0.274 | 0.298 | 0.286 | 0.0591 |
| TLR8 | rs3764880 | 0.804 | 0.232 | 0.496 | 0.262 | 0.308 | 0.299 | 0.304 | 0.0526 |
| TLR8 | rs2407992 | 0.808 | 0.393 | 0.686 | 0.906 | 0.551 | 0.532 | 0.541 | 0.0371 |
| ACE2 | rs1514283 | 0.031 | 0.001 | 0.028 | 0.300 | 0.081 | 0.086 | 0.084 | 0.0148 |
| ACE2 | rs2074192 | 0.407 | 0.454 | 0.379 | 0.319 | 0.427 | 0.419 | 0.423 | 0.0024 |
| ACE2 | rs233575 | 0.999 | 0.665 | 0.811 | 0.953 | 0.769 | 0.778 | 0.773 | 0.0171 |
| ACE2 | rs714205 | 0.561 | 0.180 | 0.375 | 0.121 | 0.185 | 0.184 | 0.184 | 0.0301 |
| ACE2 | rs4240157 | 0.971 | 0.646 | 0.763 | 0.456 | 0.616 | 0.621 | 0.618 | 0.0350 |
| ACE2 | rs4646176 | 0.029 | 0.000 | 0.023 | 0.155 | 0.043 | 0.045 | 0.044 | 0.0037 |
| ACE2 | rs4646174 | 0.968 | 0.648 | 0.767 | 0.452 | 0.621 | 0.623 | 0.622 | 0.0350 |
| ACE2 | rs879922 | 0.970 | 0.646 | 0.764 | 0.452 | 0.619 | 0.620 | 0.619 | 0.0353 |
| ACE2 | rs4646156 | 0.998 | 0.634 | 0.797 | 0.768 | 0.695 | 0.706 | 0.700 | 0.0170 |
| ACE2 | rs4646155 | 0.028 | 0.000 | 0.021 | 0.125 | 0.034 | 0.038 | 0.036 | 0.0023 |
| ACE2 | rs4646188 | 0.000 | 0.144 | 0.028 | 0.014 | 0.110 | 0.093 | 0.102 | 0.0032 |
| ACE2 | rs2048683 | 0.998 | 0.632 | 0.795 | 0.770 | 0.694 | 0.708 | 0.701 | 0.0170 |
| ACE2 | rs2285666 | 0.548 | 0.200 | 0.394 | 0.221 | 0.277 | 0.268 | 0.272 | 0.0200 |
| ACE2 | rs6632677 | 0.066 | 0.000 | 0.063 | 0.001 | 0.006 | 0.005 | 0.005 | 0.0010 |
| ACE2 | rs2106809 | 0.559 | 0.183 | 0.390 | 0.123 | 0.190 | 0.195 | 0.192 | 0.0299 |
| ACE2 | rs1978124 | 0.999 | 0.481 | 0.763 | 0.861 | 0.617 | 0.639 | 0.628 | 0.0360 |
