# Supplementary material for: Germline Genetic Variants of Viral Entry and Innate Immunity May Influence Susceptibility to SARS-CoV-2 Infection: Toward a Polygenic Risk Score for Risk Stratification
Source: Front Immunol. 2021 Mar 8;12:653489. doi: 10.3389/fimmu.2021.653489 (PMC7982482; doi:10.3389/fimmu.2021.653489)
Supplement: Supplementary file 3 [file Table_3.DOCX]

Supplementary Material

# Supplementary Table 3

**Risk allele frequencies (RAFs) of the eight lead COVID-19 related SNPs (**[**1**](#_ENREF_1)**) in different populations** Data presented here were downloaded from the gnomAD database (gnomad.broadinstitute.org). Odds ratios (ORs) demonstrated are according to ([1](#_ENREF_1)). RAFs in East Asian (EAS), Non-Finnish European (NFE), Latino/Admixed American (AMR) and African/African American (AFR) superpopulations are presented.

| SNP | risk allele | alternative allele | OR | RAF | | | | |
| --- | --- | --- | --- | --- | --- | --- | --- | --- |
|  |  |  |  | ALL | EAS | NFE | AMR | AFR |
| rs73064425 | T | C | 2.1 | 0.060 | 0.001 | 0.075 | 0.050 | 0.013 |
| rs9380142 | A | G | 1.3 | 0.728 | 0.641 | 0.684 | 0.752 | 0.804 |
| rs143334143 | A | G | 1.9 | 0.084 | 0.047 | 0.081 | 0.116 | 0.050 |
| rs3131294 | G | A | 1.5 | 0.912 | 0.996 | 0.863 | 0.932 | 0.973 |
| rs10735079 | A | G | 1.3 | 0.700 | 0.775 | 0.646 | 0.736 | 0.775 |
| rs2109069 | A | G | 1.4 | 0.274 | 0.133 | 0.314 | 0.251 | 0.220 |
| rs74956615 | A | T | 1.6 | 0.035 | 0.000 | 0.053 | 0.032 | 0.012 |
| rs2236757 | A | G | 1.3 | 0.357 | 0.581 | 0.303 | 0.463 | 0.213 |

Reference

1. Pairo-Castineira E, Clohisey S, Klaric L, Bretherick AD, Rawlik K, Pasko D, et al. Genetic mechanisms of critical illness in Covid-19. Nature. 2020.
